# Effects of Aerobic Exercise and Mind-Body Exercise in Parkinson’s Disease: A Mixed-Treatment Comparison Analysis

**DOI:** 10.3389/fnagi.2021.739115

**Published:** 2021-11-18

**Authors:** Chunxiao Wu, Yingshan Xu, Hongji Guo, Chunzhi Tang, Dongfeng Chen, Meiling Zhu

**Affiliations:** ^1^Shenzhen Hospital of Integrated Traditional Chinese and Western Medicine, Guangzhou University of Chinese Medicine, Guangdong, China; ^2^The Research Center of Basic Integrative Medicine, Guangzhou University of Chinese Medicine, Guangzhou, China; ^3^Clinical Medical of Acupuncture, Moxibustion and Rehabilitation, Guangzhou University of Chinese Medicine, Guangzhou, China

**Keywords:** aerobic exercises, mind-body exercise, Parkinson’s disease, network meta-analysis, dose response

## Abstract

**Background/Objectives:** Aerobic exercise and mind-body exercise, are vital for improving motor and non-motor functional performance of Parkinson’s disease (PD). However, evidence-based recommendations on which type of exercise is most suitable for each individual are still lacking. Therefore, we conduct a network meta-analysis to assess the relative efficacy of aerobic and mind-body exercise on motor function and non-motor symptoms in Parkinson’s disease and to determine which of these therapies are the most suitable.

**Design:** A network meta-analysis and dose-response analysis.

**Setting and Participants:** Medline, Embase (all via Ovid), and the Cochrane Central Register of Controlled Trials were comprehensively searched for related trials through April 2021.

**Measurements:** Study quality was evaluated using the Cochrane Risk of Bias Tool. The effect sizes of continuous outcomes were calculated using mean differences (MDs) or standardized mean differences (SMDs). A network meta-analysis with a frequentist approach was conducted to estimate the efficacy and probability rankings of the therapies. The dose-response relationship was determined based on metaregression and SUCRA.

**Results:** Fifty-two trials with 1971 patients evaluating six different therapies were identified. For the UPDRS-motor score and TUG score, yoga all ranked highest (SUCRA = 92.8%, 92.6%, respectively). The SUCRA indicated that walking may best improve the BBS score (SUCRA = 90.2%). Depression, cognitive and activities of daily living scores were significantly improved by yoga (SUCRA: 86.3, 95.1, and 79.5%, respectively). In the dose-response analysis, 60-min sessions, two times a week might be the most suitable dose of yoga for reducing the UPDRS-motor score of PD patients.

**Conclusion:** Yoga and walking are important options for increasing functional mobility and balance function, and yoga might be particularly effective for decreasing depressive symptoms and cognitive impairment and improving activities of daily living in PD. The potential optimal dose of yoga for enhancing motor ability in PD patients is 60-min sessions, two times a week.

**Registration:** PROSPERO CRD42021224823.

## Introduction

According to epidemiological studies of Parkinson’s disease (PD), the global PD population reached approximately 6,100,000 in 2016 (Collaborators, 2018, 2019), making it the second most common neurodegenerative disorder. PD affects the motor and non-motor systems, resulting in poor quality of life for patients and a heavy burden on families and society. There is no curative treatment for PD, which poses a great challenge for global health systems that needs to be resolved.

Although pharmacotherapy is currently still the first-line treatment, side effects and response fluctuations limit its application (Connolly and Lang, 2014; Ellis and Fell, 2017; Stoker and Barker, 2020). The latest guideline (Canadian guideline for Parkinson’s disease) and studies have indicated that physical exercise, especially aerobic and mind-body exercise, are vital for improving motor and non-motor functional performance and delaying the progression of PD (Ahlskog, 2018; Schenkman et al., 2018; Grimes et al., 2019; van der Kolk et al., 2019; Deuel and Seeberger, 2020). Previous meta-analyses have demonstrated that these types of exercise may be beneficial for maintaining brain health, promoting functional mobility performance, psychosocial function and quality of life (Shu et al., 2014; Kwok et al., 2016; Chen et al., 2020; Schootemeijer et al., 2020). Moreover, these types of exercise might exert positive effect on cognitive function and wellbeing by enhancing cerebrovascular angiogenesis and regulating the brain plasticity (da Silva et al., 2018; Mandolesi et al., 2018; Pianta et al., 2019). However, aerobic exercise and mind-body exercise includes various types of exercise, such as treadmill exercise, walking, cycling, dance, tai chi and yoga. Combining different interventions when conducting a meta-analysis may induce confounding factors. Moreover, evidence-based recommendations on which type of exercise is most suitable for each individual are still lacking. Therefore, systematically determining the most effective treatment options for particular signs and symptoms from among all available types of aerobic exercise and mind-body exercise is critical to providing individual evidence-based recommendations for PD patients.

Network meta-analysis is a technique that uses direct and indirect results to compare multiple interventions simultaneously and estimates the rank order of the treatments, providing evidence-based recommendations for assisting medical decision making (Rouse et al., 2017; Dias and Caldwell, 2019). Therefore, in this study, we conducted a network meta-analysis to evaluate the effect of aerobic and mind-body exercise on motor function, functional mobility, psychosocial status and activities of daily living. Furthermore, we also explored the rankings of different exercise treatments and provided evidence-based recommendations for patients with PD under different situations.

## Materials and Methods

### Study Registration

This network-meta analysis was prospectively registered in the International Prospective Register of Systematic Reviews (PROSPERO) with a record number CRD42021224823.

### Search Strategy

The electronic databases Medline, Embase (all via Ovid), and the Cochrane Library were searched for all relevant citations published from inception through April 3, 2020. We used various combinations of medical subject headings and free terms, which included Parkinson’s disease, aerobic and mind-body exercise (a list of relevant exercise interventions), and randomized clinical trial designs (search strategies are listed in Supplementary 1).

### Selection and Exclusion Criteria

Eligible studies were included when they met the following criteria: (1). All studies were randomized controlled trials (RCTs). (2) The population of the included studies was adult patients diagnosed with PD. (3) Interventions contained at least one of the following exercises: treadmill exercise, walking, cycling, dance, yoga, and tai chi. The control group received usual care, was a waitlist control or performed other non-aerobic and non-mind-body exercise. (4) Outcomes of the studies included at least one of the following measurements: Unified Parkinson’s Disease Rating Scale (UPDRS) score [UPDRS or Movement Disorder Society-Unified Parkinson’s disease rating scale scores (MDS-UPDRS)], Berg Balance Scale (BBS) score, Timed-Up-and-Go (TUG) score, psychosocial outcomes (depressive scale: BDI, HADS-depression; cognitive functional scale: DRS, MMSE, MoCA, PDQ39-cognition, and UPDRS-mental) and activities of daily living (ADL) (UPDRS-ADL, PDQ39-ADL).

Studies were excluded if insufficient data or information concerning assessment was provided. We also excluded quasi-RCTs, animal trials, clinical protocols, conference abstracts, case reports and systematic reviews.

### Data Extraction and Quality Assessment

Two investigators (YX & HG) independently extracted the relevant data and information from the eligible studies. Basic information about the study characteristics (first author, year, design), population, interventions and comparisons (duration, frequency, length), and measurement outcomes was extracted. Another two reviewers (CW & CT) evaluated the quality of the included studies based on the standard criteria of the Cochrane Risk of Bias Tool (Savovic et al., 2014). Any disagreements that existed were resolved through discussion. If necessary, a senior investigator (MZ) was consulted to achieve a consensus.

### Outcomes

The outcomes of this network meta-analysis were listed as follow:

#### Primary Outcome

Motor outcomes: UPDRS-motor score [assessed by UPDRS III and MDS-UPDRS III, UPDRS III were converted to MDS-UPDRS III by adding 7 points according to the validated calibration method (Hentz et al., 2015)]; BBS and TUG outcomes.

Non-motor outcomes: Psychosocial outcomes (depressive and cognitive functional scale scores) and ADL.

#### Secondary Outcome

Safety outcomes: Non-serious and serious adverse events.

### Statistical Analysis

We performed a pairwise meta-analysis first. All variables were continuous data and presented as mean with standard deviation (SD). Mean differences (MD = Absolute difference between the mean value in two groups, defined as the difference in means between treatment and control group and was calculated using the same scale) or standardized mean differences (SMD = Difference in mean outcome between groups/Standard deviation of outcome among participants, which was used to combine data when trials with different scale) with 95% confidence interval (CI) was reported as a continuous outcome (Borenstein et al., 2010; Cumpston et al., 2019). We assessed heterogeneity by testing the *I*^2^ statistic. If statistically quantifiable heterogeneity existed, a random-effects model was fitted. Otherwise, we used a fixed-effect model (Borenstein et al., 2010).

Then, we conducted a network meta-analysis for each outcome using a frequentist approach and the netmeta command in Stata version 14 (Shim et al., 2017). We quantified potential inconsistencies between the direct and indirect results by using network side-split analysis and a design-by-treatment interaction model (Dias et al., 2010; White et al., 2012). If the *p*-value of the design-by-treatment interaction model and the network side-split analysis exceeded 5%, a consistency model was used to evaluate the effect size of the multiple treatment comparisons.

We estimated the ranking probabilities of each treatment for different outcomes based on the surface under the cumulative ranking curve (SUCRA) and mean ranking (Salanti et al., 2011). Moreover, to examine the stability of the result and evaluate whether the results were impacted by study characteristic, sensitivity analysis was conducted based on the baseline patient characteristics. We also explored the potential dose-response relationship using metaregression if the data of the included studies allowed it. To assess publication bias, we used the comparison-adjusted funnel plot to detect the risk of potential publication bias. If the effect size of the included studies was distributed symmetrically, which indicated that there was minimal publication bias in this network meta-analysis.

## Results

### Study Identification and Selection

We retrieved a total of 5,919 publications from the electronic databases and eliminated 1,592 duplicate publications. A total of 4,327 publications were left for screening according to their titles and abstracts. Of those, 4,095 publications were removed, and 232 remaining publications were then identified as potentially eligible studies and underwent a full-text review. Finally, fifty-two relevant publications were included in the network meta-analysis (Supplementary Figure 1).

### Characteristics of the Included Studies

A total of 52 eligible RCTs with 1,971 patients diagnosed with PD were included in this network meta-analysis. The interventions of the included trials mainly included treadmill exercise, walking, cycling, dance, yoga, and tai chi. Most trials used usual care, waitlist or other non-aerobic exercise as the control. The duration of each intervention varied from 20 to 90 min per session, and the frequency ranged from one to five times per week. Forty-two RCTs assessed motor function using the UPDRS-motor score. Fourteen studies used the BBS to assess balance function. Twenty-three studies evaluated mobility function using the TUG test, twelve RCTs assessed depressive outcomes, 13 RCTs evaluated cognition, and 17 studies reported ADL outcomes (see Supplementary Table 1).

### Quality Assessment of the Included Studies

Among the 52 RCTs, 65.38% had a low risk in terms of random sequence generation, and 63.46% reported the use of allocation concealment methods. Few studies (11.54%) used blinding methods for participants and personnel because exercise is a non-pharmacologic treatment. Forty studies (77.36%) reported a low risk for bias in terms of blinding the outcome assessment. RCTs (84.62%) had a low risk of attrition bias. Forty-two studies reported a low risk of reporting bias. Overall, 67.31% were deemed to have a low risk of poor methodological quality, whereas 17 studies were regarded as having poor methodological quality (Supplementary Figures 2,3).

### Analysis of Outcomes

#### Primary Outcomes

##### Motor Outcomes

###### Unified Parkinson’s Disease Rating Scale-Motor

Forty-two RCTs with six different therapy categories assessed motor function using UPDRS III-motor measurements. Treadmill exercise contributed 18.8% to the network plot, walking 8.2%, cycling 4.7%, dance 9.4%, yoga 5.8%, tai chi 7%, and the control group 45.8% (Supplementary Figure 4A).

**FIGURE 1:**
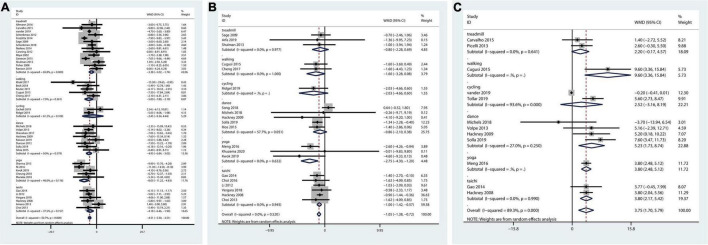
Pairwise meta-analysis of aerobic and mind-body therapies on motor outcomes. **(A)** UPDRS-motor outcome. **(B)** TUG outcome. **(C)** BBS outcome. Meta-analysis results for pair-wise comparisons represented by MD and 95% credible interval (CrI).

The pairwise meta-analysis illustrated that all therapies could decrease the overall UPDRS motor score. Those performing treadmill exercise, walking, dance, yoga, or tai chi all functioned better than those in the control group in terms of the UPDRS-motor score (Figure 1A).

Quantification of the inconsistencies between direct and indirect comparisons using node-splitting methods and the design-by-treatment interaction model showed that all *p*-values exceeded 0.05 (Supplementary Table 2), which indicated satisfactory consistency.

**FIGURE 2:**
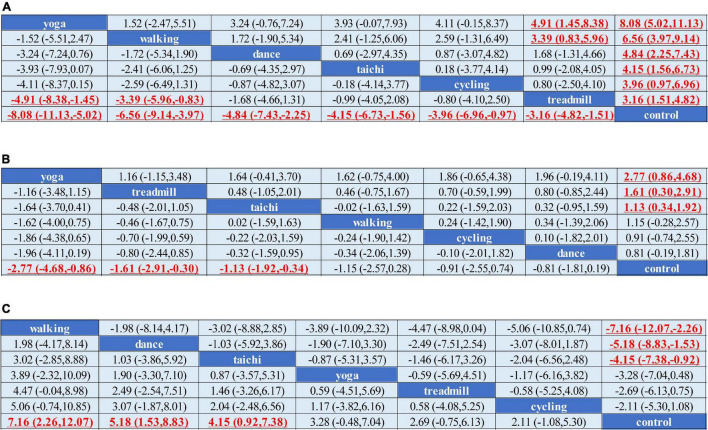
Network meta-analysis of the efficacy of exercise therapies on motor outcomes. **(A)** UPDRS-motor outcome. **(B)** TUG outcome. **(C)** BBS outcome. MD and 95% credible interval (CrI) estimations were calculated as column-defining interventions compared with row-defining interventions. Significant results were labeled with bold, red and underlined.

**FIGURE 3:**
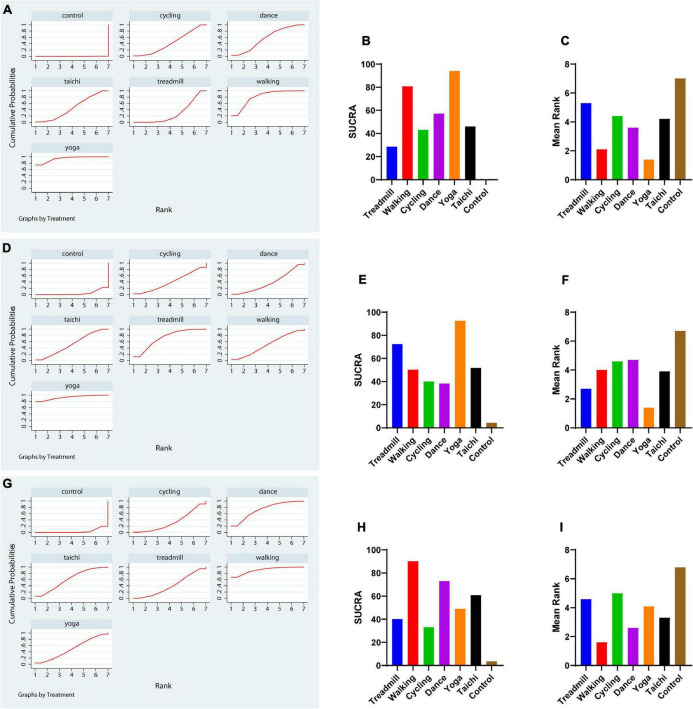
The rank probability of various interventions based on the SUCRA. **(A–C)** Rank probability and mean rank on UPDRS-motor outcome. **(D–F)** TUG outcome. **(G–I)** BBS outcome. The greater the SUCRA value is, the better the rank.

Network meta-analysis indicated that treadmill exercise [MD = –3.23,CI = (–4.80, –1.67)], walking [MD = –6.12, CI = (–8.62, –3.61)], dance [MD = –4.84, CI = (–7.45, –2.24)], yoga [MD = –8.07, CI = (–11.14, –5.00)], and tai chi [MD = –4.66, CI = (–7.10, –2.22)] were superior to the control in reducing the UPDRS-motor score. Yoga and walking were significantly more effective in decreasing UPDRS-motor scores than treadmill therapy (Figure 2A). The ranking probability of six different interventions illustrated that yoga (SUCRA:94.1%) ranked first for the UPDRS-motor score based on the SUCRA, followed by walking, dance, cycling, tai chi, and treadmill therapy (Figures 3A–C).

##### Dose-Response Analysis

**FIGURE 4:**
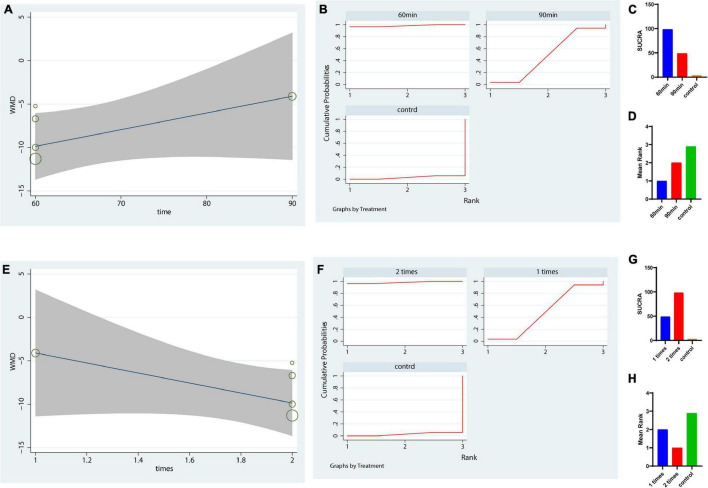
Dose response of yoga using meta-regression and SUCRA on UPDRS-motor outcome. **(A–D)** The potential optimal time of yoga per session in UPDRS motor scale; **(E–H)** The potential optimal frequency of yoga each week in UPDRS motor outcome. **(A,E)** Dose-response curve for yoga using metaregression. **(B–D,F–H)** Dose response of yoga based on the rank probability and mean rank. The greater the SUCRA value is, the better the rank.

Because yoga was ranked first for the UPDRS-motor score, we further analyzed the optimal dose of yoga therapy for improving motor function in PD patients. We analyzed the potential optimal time of yoga per session in UPDRS motor score. Our metaregression revealed that the effect size was not significantly increased with increasing yoga intervention time each session (*p* = 0.15 > 0.05, Figure 4A). The results showed that a duration of 60 min (each time) was the most suitable duration for reducing the UPDRS-motor score of PD patients (SUCRA: 98.2%, Figures 4B–D). Besides, the result also indicated that 2 times a week might be the most suitable frequency for reducing the UPDRS-motor score of PD patients (SUCRA: 98.2%, Figures 4E–H).

###### Timed-Up-and-Go Test

The network plot of TUG outcome was shown in Supplementary Figure 4B. And the pairwise meta-analysis showed that compared with the control, yoga and tai chi therapies significantly decreased the TUG score. Overall therapies could reduce the TUG score (Figure 1B).

The consistency tests between the direct and indirect effects all indicated that the *p*-value exceeded 0.05 (Supplementary Table 2).

We conducted a network meta-analysis of TUG outcomes, and the results illustrated that yoga (MD = –2.77, CI = –4.68, –0.86), tai chi (MD = –1.13, CI = –1.92, –0.34), and treadmill exercise (MD = –1.61, CI = –2.91, –0.30) were more beneficial in reducing the TUG score than the control (Figure 2B). Moreover, we evaluated the rankings of the various treatments based on TUG scores and found that yoga ranked the highest (SUCRA: 92.6%), followed by treadmill exercise, tai chi, walking, cycling and dance (Figures 3D–F).

###### Berge Balance Scale

Fourteen studies including six treatments were included in the network meta-analysis evaluating BBS outcomes (Supplementary Figure 4C). Figure 1C shows that therapies could enhance the overall balance ability of PD patients. Walking, dance, yoga, and tai chi were highly effective in increasing balance ability.

The comparisons indicated satisfactory consistency (all, *p* > 0.05, Supplementary Table 2). The network comparison revealed that walking (MD = 7.16, CI = (2.26, 12.07), dance [MD = 5.18, CI = (1.53, 8.83)], and tai chi [MD = 4.15, CI = (0.92, 7.38)] were superior to the control in improving the BBS score (Figure 2C). The SUCRA indicated that walking had the highest rank for the BBS score (SUCRA: 90.2%), followed by dance, tai chi, and yoga (Figures 3G–I).

##### Clustered Ranking Plot of the Network

**FIGURE 5:**
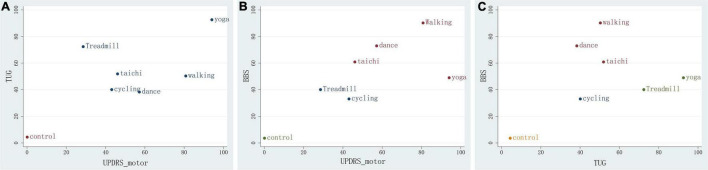
Clustered ranking plot of the network. **(A)** For UPDRS-motor&TUG outcomes. **(B)** For UPDRS&BBS. **(C)** For TUG&BBS outcomes. The plots are based on cluster analyses of SUCRA values. Each plot shows the SUCRA values for two different outcomes. The therapies in the upper right corner are more effective than the other therapies.

We constructed a clustered ranking plot of the network (UPDRS-motor and TUG, UPDRS-motor and BBS, and TUG and BBS) to comprehensively evaluate the most suitable treatment for improving motor function in PD. The results showed that the yoga, walking, dance, and tai chi groups had higher SUCRA values in the clustered ranking plot, which indicated that yoga and walking in particular, in addition to dance and tai chi, are the most suitable therapies for increasing overall motor function in PD patients (Figure 5).

##### Non-motor Outcomes

###### Depression Scale

Twelve studies with five treatments assessed depression scales (Supplementary Figure 4D). The pairwise meta-analysis showed that treadmill and yoga therapy significantly decreased depressive symptoms in the PD group compared with the control group (Supplementary Figure 5A).

All *p*-values exceeded 0.05 in the network side-split model, which revealed that the direct and indirect comparison outcomes were consistent (Supplementary Table 2).

The network meta-analysis of five different exercises based on depression scale outcomes showed that yoga was more effective in decreasing depressive symptoms than the control [SMD = –0.88, CI (–1.64, –0.12)]. Yoga, treadmill exercise and dance were significantly superior to cycling for reducing depression scores. Yoga therapy (SUCRA: 86.3%) was ranked highest for ameliorating PD with depression, followed by walking, treadmill exercise and dance (Supplementary Figures 6A, 7A and Supplementary Table 3).

###### Cognitive Assessment

Thirteen studies with five interventions assessed cognitive function (Supplementary Figure 4E). The pairwise meta-analysis showed that only dance therapy was more effective in improving the cognition of PD patients than the control (Supplementary Figure 5B).

We found that all *p-*values of consistency tests were higher than 0.05, which indicated that direct and indirect effects had good consistency (Supplementary Table 2).

The results of the network meta-analysis showed that only yoga was associated with significantly higher cognitive function scores than the control [SMD = 1.32 (0.11, 2.54)]. The ranks of the five interventions for enhancing cognitive function in PD were as follows: yoga, dance, treadmill exercise, walking, and cycling (Supplementary Figures 6, 7B and Supplementary Table 3).

###### Activities of Daily Living

Seventeen studies assessed ADL in PD. We conducted a pairwise meta and the results demonstrated that treadmill exercise, cycling and yoga were all associated with significantly greater changes in ADL scores than the control (Supplementary Figure 5C).

The *p*–values of consistency tests were substantially higher than 0.05, which demonstrated that the network analysis had good consistency (Supplementary Table 2).

We conducted a network meta-analysis to assess the intervention effects in terms of ADL. The results revealed that yoga, cycling, and treadmill exercise were all superior to the control in improving ADL [SMD = –0.60 (–1.00, –0.20); SMD = –0.53 (–0.97, –0.09); and SMD = –0.47 (–0.74, –0.19), respectively]. Furthermore, yoga and treadmill therapy were significantly more effective in improving performance of activities of daily living than dance. The rankings of these five interventions, which were based on the SUCRAs for the ADL scores, showed that yoga ranked first, followed by cycling, treadmill exercise and tai chi (Supplementary Figures 6C, 7C and Supplementary Table 3).

#### Secondary Outcomes

##### Adverse Outcomes

Thirty-three studies reported adverse events related to exercise therapies. Among these RCTs, 21 studies reported that no adverse events occurred during treatment. The remaining studies reported a minimal number of adverse events, mainly knee, neck and back pain, muscle soreness and non-injurious falls. All these events and the symptoms were resolved by resting or simple treatment without further management (see Supplementary Table 1).

##### Sensitivity Analysis

In order to minimize the influence of heterogeneous baseline severity of PD in our study, we restricted our analysis into trials with early to-moderate stage of Parkinson’s disease as sensitivity analysis. The result showed that five therapies were superior to the control in reducing the UPDRS-motor score and yoga ranked first based on the SUCRA (93.4%). Besides, a sensitivity analysis after excluding studies that contained resistance active exercise as control group was conducted. All therapies except cycling were significantly superior to placebo and yoga also ranked first based on the SUCRA (95.2%), which were consistent with those previous produced (Supplementary Figures 8A–H). Besides, the sensitivity analysis of other outcomes (TUG, BBS, Depression scale, Cognitive outcome and ADL) were also in line with those previous conducted (the specific analysis were shown in Supplementary Figures 8I–X and Supplementary Figures 9A–H). The sensitivity analysis all indicated the results were stable.

##### Publication Bias

We constructed a funnel plot of our outcomes to assess publication bias. The plots all showed that the effect size of the included studies was distributed symmetrically, which demonstrated that there was minimal publication bias in this analysis (Supplementary Figure 10).

## Discussion

### Principal Findings

We conducted a network meta-analysis that included 52 RCTs with six different treatments and obtained several principal findings summarized as follows.

The pooled results suggested that yoga, walking, dance, and tai chi were superior in reducing the UPDRS-motor scores of PD patients, and yoga and walking might be highly effective based on the SUCRA rankings. In the TUG test, yoga, tai chi and treadmill exercise were superior to the control. Additionally, yoga ranked first in decreasing the TUG score. Moreover, in terms of the BBS Walking had the highest efficacy among these various treatments in terms of BBS score. According to the clustered ranking plot of the network, yoga and walking might be the important therapies for comprehensively increasing motor and balance function in PD patients. Previous studies also found that yoga and walking exerted beneficial effects on motor function in PD (Bombieri et al., 2017; Cugusi et al., 2017; Godi et al., 2019; Adams et al., 2020; De Santis and Kaplan, 2020; Deuel and Seeberger, 2020).

Regarding psychosocial outcomes, the results suggested that yoga and dance could be significantly more effective in reducing depressive symptoms and improving cognitive function. Overall, yoga might be the important alternative therapy for PD patients with depression or dementia according to the SUCRA rankings. These findings were in line with previous studies revealing that patients report that yoga is enjoyable, feasible and beneficial regarding their depressive and anxiety symptoms (Jin et al., 2019; Deuel and Seeberger, 2020; Sagarwala and Nasrallah, 2020). In addition, a previous study demonstrated that yoga enhanced executive functions, memory and attention to some extent, which was consistent with our results (Chobe et al., 2020). Furthermore, dance might also be an important option that could be recommended for PD patients with cognitive impairment because the pairwise-meta result and previous studies all indicated that dance has potential benefit for ameliorating cognitive dysfunction (Kalyani et al., 2019; Hasan et al., 2021).

For the ADL scale, we found that yoga, cycling, and treadmills were more effective than controls in improving the ADL of PD patients, especially yoga, which ranked higher in the ADL assessment, suggesting that yoga might be the vital option for patients with poor activities of daily living. A previous review also agreed with our results and indicated that yoga therapy improved the PDQ-ADL score and mood of PD patients more than other therapies (Subramanian, 2017).

No severe adverse events occurred during exercise therapies. Only a few patients suffered muscle soreness or minor low back or knee pain. All these events can be resolved by resting and not affect the treatment process. Overall, aerobic or mind-body therapy is a safer treatment for addressing PD symptoms than other therapies.

With regard to the quality of the included RCTs, 67.31% were determined to be high-quality RCTs, which indicated that the pooled results were robust and reliable overall. However, the high risk of improper blinding of participants still existed in this analysis, which might decrease the strength of the evidence to a degree.

### Findings in Relation to Previous Reviews

To our knowledge, this network meta-analysis is the first study to include all previously examined types of aerobic and mind-body exercise and to explore the rankings of various types of therapies for PD according to a comprehensive range of outcomes. Most of the previous meta-analyses focused on evaluating the efficacy of only one type of exercise for PD (Cugusi et al., 2017; Liu et al., 2019; Robinson et al., 2019; Carapellotti et al., 2020). However, head-to-head comparisons between various therapies are still lacking, making it difficult for patients to make optimal decisions. Previous reviews about aerobic exercise reported that aerobic exercise have beneficial effects in improving motor action, balance function in PD patients (Shu et al., 2014; Schootemeijer et al., 2020), which were agreed with our results of aerobic exercise. A review compared the efficacy of different kinds of mind-body exercise, including yoga, and tai chi, for motor outcomes, depressive symptoms and quality of life in PD and implied that mind-body exercise might have significant improvements in motor function, depressive symptoms and quality of life in Parkinson’s disease, which was consistent with most of our results (Jin et al., 2019). Another review also investigated the effect of mind-body exercises (including yoga, taichi, and dance) on physiological outcomes for PD, the result indicated that mind-body exercises have moderate to large beneficial effects on motor function (Kwok et al., 2016). However, these studies did not examine all previously examined types of aerobic and mind-body exercise together that might prohibited accurate comparisons of the efficacy of aerobic and mind-body therapies for PD outcomes. Furthermore, the assessment of non-motor function, especially psychiatric symptoms, was not amply investigated in these studies. Therefore, our analysis comprehensively assesses the efficacy of all previously examined aerobic and mind-body exercises for treating PD and determines the important therapy for improving motor and non-motor outcomes in PD.

### Implications for Clinical Practice

Overall, our results have several clinical implications. First, the results suggest that yoga and walking may be the important exercises for enhancing motor function and balance functional ability. Second, yoga as a mind-body exercise is a good option for PD complicated with depressive disorder and cognitive impairment. Moreover, yoga therapy for 60 min, two times a week can be recommended as the potential optimal dose for improving mobility in PD. Therefore, these evidence-based results could be recommended for PD patients and assist clinical doctors in making appropriate decisions.

### Limitations

Our analysis has several limitations. In terms of non-motor outcomes, few studies and interventions were included in the analysis. Moreover, RCTs of head-to-head comparisons with other active therapies that included non-motor assessments were lacking, providing little direct evidence for overall or accurate evaluation of the efficacy of exercise therapies for non-motor symptoms (especially depressive symptoms and cognitive function) in PD. Besides, because of the limit number of yoga studies and the limitations of SUCRA, the results in this study should be interpreted with caution. Hence, more studies involving yoga are needed. In addition, most of the included studies lacked participant blinding because the exercise therapies were non-drug interventions. However, this could have resulted in bias for the intervention group.

## Conclusion

The network meta-analysis reported herein indicates that aerobic and mind-body therapies significantly improve the motor function of PD patients at all stages. Yoga and walking could be prior to recommend for PD patients with motor symptoms and balance impairment. Yoga may also be the important option for decreasing depressive disorder and cognitive impairment and could be recommended as part of treatment for life style changes in patients with PD. The potential appropriate dose of yoga for enhancing motor ability in PD patients is 60-min sessions, 2 times a week.

## Data Availability Statement

The original contributions presented in the study are included in the article/Supplementary Material, further inquiries can be directed to the corresponding author/s.

## Author Contributions

MZ and DC designed the study and revised the manuscript for important intellectual content. YX and HG acquired the data. CW and CT analyzed and interpreted the data. CW drafted the manuscript. All authors read and approved the final manuscript.

## Conflict of Interest

The authors declare that the research was conducted in the absence of any commercial or financial relationships that could be construed as a potential conflict of interest.

## Publisher’s Note

All claims expressed in this article are solely those of the authors and do not necessarily represent those of their affiliated organizations, or those of the publisher, the editors and the reviewers. Any product that may be evaluated in this article, or claim that may be made by its manufacturer, is not guaranteed or endorsed by the publisher.
