# Supplementary material for: Effects of Aerobic Exercise and Mind-Body Exercise in Parkinson’s Disease: A Mixed-Treatment Comparison Analysis
Source: Front Aging Neurosci. 2021 Nov 18;13:739115. doi: 10.3389/fnagi.2021.739115 (PMC8637737; doi:10.3389/fnagi.2021.739115)
Supplement: Supplementary file 1 [file Data_Sheet_1.docx]

**Supplementary Online Content**

[Supplementary 1. Search strategy for each database 1](#_Toc83117139)

[Supplementary Table 1. Detailed information of included studies. 4](#_Toc83117140)

[Supplementary Table 2. Assessment of inconsistency 15](#_Toc83117141)

[Supplementary Table 3. Mean rank and SUCRA 17](#_Toc83117142)

[Supplementary Figure 1. The flowchart of screening process 18](#_Toc83117143)

[Supplementary Figure 2. Risk of bias summary 19](#_Toc83117144)

[Supplementary Figure 3. Risk of bias graph 20](#_Toc83117145)

[Supplementary Figure 4. Network plot 20](#_Toc83117146)

[Supplementary Figure 5. Results of meta-analysis of direct comparison 21](#_Toc83117147)

[Supplementary Figure 6. Results of network meta-analysis 22](#_Toc83117148)

[Supplementary Figure 7. Rank plot: cumulative rank of varied treatments for PD in non-motor outcomes. 23](#_Toc83117149)

[Supplementary Figure 8. Sensitivity analysis of motor outcomes 23](#_Toc83117150)

[Supplementary Figure 9. Sensitivity analysis of non-motor outcomes 25](#_Toc83117151)

[Supplementary Figure 10 Comparison-adjusted funnel plot for the network of the outcomes. 27](#_Toc83117152)

[Supplementary references 28](#_Toc83117153)

# Supplementary 1. Search strategy for each database

**MEDLINE (**OVID)

1. Parkinson.tw.
2. Parkinson$.tw.
3. (PD or IPD).tw.
4. (Parkinson$ adj5 Diseas$).tw.
5. exp Parkinson Disease/

6. or/1-5

7. exp Exercise/

8. aerobic.mp.

9. (aerobic$ adj train$).tw

10. ((aerobic$ or isometric$) adj2 exercise$).tw

11. physical activity.mp.

12. exp walking/

13. walking$.tw.

14. exp jogging/

15. jogging$.tw.

16. exp swimming/
17. swimming$.tw.
18. exp Bicycling/
19. (bicycling$ or cycling$).tw.

20. Treadmill.tw.

21. exp TAI JI/
22. (tai chi or tai ji or ji quan tai).tw.
23. exp YOGA/
24. yoga.tw

25. exp dancing/

26. danc$.tw.

27. 7 or 8 or 9 or 10 or 11 or 12 or 13 or 14 or 15 or 16 or 17 or 18 or 19 or 20 or 21 or 22 or 23 or 24 or 25 or 26

28.randomised controlled trial.pt.
29. controlled clinical trial.pt.
30. randomized.ab.
31. randomly.ab.
32. trial.ab.
33. 28 or 29 or 30 or 31 or 32
34. animals.sh.

35. 33 not 34

36. 6 and 27 and 35

**Embase (OVID)**

1. Parkinson.tw.
2. Parkinson$.tw.
3. (PD or IPD).tw.
4. (Parkinson$ adj5 Diseas$).tw.
5. exp Parkinson Disease/

6. or/1-5

7. exp Exercise/

8. aerobic.mp.

9. (aerobic$ adj train$).tw

10. ((aerobic$ or isometric$) adj2 exercise$).tw

11. physical activity.mp.

12. exp walking/

13. walking$.tw.

14. exp jogging/

15. jogging$.tw.

16. exp swimming/
17. swimming$.tw.
18. exp Bicycling/
19. (bicycling$ or cycling$).tw.

20. Treadmill.tw.

21. exp TAI JI/
22. (tai chi or tai ji or ji quan tai).tw.
23. exp YOGA/
24. yoga.tw

25. exp dancing/

26. danc$.tw.

27. 7 or 8 or 9 or 10 or 11 or 12 or 13 or 14 or 15 or 16 or 17 or 18 or 19 or 20 or 21 or 22 or 23 or 24 or 25 or 26

28. "randomi?ed controlled trial".mp. [mp=title, abstract, subject headings, heading word, drug trade name, original title, device manufacturer, drug manufacturer, device trade name, keyword]

29. "controlled clinical trial".mp. [mp=title, abstract, subject headings, heading word, drug trade name, original title, device manufacturer, drug manufacturer, device trade name, keyword]

30. random*.mp.

31. randomised controlled trial/

32. or/28-31

33. 6 and 27 and 32

**Cochrane Central Register of Controlled Trials**

#1 (PD or IPD)

#2 (Parkinson? next/5 Diseas?)

#3 MeSH descriptor: [Parkinson Disease] explode all trees

#4 #1 or #2 or #3

#5 MeSH descriptor: [Exercise Therapy] explode all trees

#6 "Physical therap*"
#7 "physical activit*"
#8 exercis*
#9 aerobic
#10 "physical* fit*"
#11 "physical capacity"
#12 "physical training"
#13 walk* or treadmill

#14 jogging
#15 Cycling
#16 swim*
#17 danc*
#18 yoga
#19 "tai chi"
#20 #5 or #6 or #7 or #8 or #9 or #10 or #11 or #12 or #13 or #14 or #15 or #16 or #17 or #18 or #19

#21 #4 and #20

# Supplementary Table 1. Detailed information of included studies.

| **Author** | **year** | **Study Population** | **Age(y, mean**  **±SD)** | **Gender, N male (%)** | **Ethnicity** | **Intervention** | **Control1** | **Control2** | **Outcome measures** | **Change from the baseline**  **UPDRS-motor**  **(mean±SD)** | | **Change from the baseline TUG(mean±SD)** | | **Change from the baseline**  **BBS(mean±SD)** | | **Change from the baseline**  **Depression**  **(mean±SD)** | | **Change from the baseline**  **Cognitive(mean±SD)** | | **Change from the baseline**  **ADL(mean±SD)** | | **Adverse events** |
| --- | --- | --- | --- | --- | --- | --- | --- | --- | --- | --- | --- | --- | --- | --- | --- | --- | --- | --- | --- | --- | --- | --- |
|  |  |  |  |  |  |  |  |  |  | **treatment** | **control** | **treatment** | **control** | **treatment** | **control** | **treatment** | **control** | **treatment** | **control** | **treatment** | **control** |  |
| Altmann(Altmann et al., 2016) | 2016 | Parkinson disease  Hoehn & Yahr1-3 | Group 1:  62.8(8.6)  Group 2:  63.3(7.3)  Group 3:  67.8(9.8) | NR | NR | treadmill | Balance | no-contact control | MDS-UPDRS-  motor; BDI;  DRS; ADL | -0.9±8.33 | 2.1±7.46 | NA | NA | NA | NA | -0.7±5.35 | 3.4±4.73 | -1.3±2.61 | 0.1±4.27 | 2.1±4.06 | 2.2±3.82 | NR |
|  |  |  |  |  |  | Duration:20min to 45min |  |  |  |  |  |  |  |  |  |  |  |  |  |  |  |  |
|  |  |  |  |  |  | freq: 3 times a week |  |  |  |  |  |  |  |  |  |  |  |  |  |  |  |  |
|  |  |  |  |  |  | length of intervention:  16weeks |  |  |  |  |  |  |  |  |  |  |  |  |  |  |  |  |
| Carvalho(Carvalho et al., 2015) | 2015 | Parkinson disease  Hoehn & Yahr1-3 | Group 1:  64.8(11.9)  Group 2:  64.1(9.9)  Group 3:  62.1(11.7) | Group 1:  4(80)  Group 2:  6(71.4)  Group 3:  5(55.6) | NR | treadmill | control(strength training) | control(physiotherapy) | UPDRS-motor;  BBS;  ADL | -10.8±8.67 | -1±14.72 | NA | NA | 1.4±4.07 | 0±3.16 | NA | NA | NA | NA | -6.4±5.74 | 1±8.76 | no adverse event |
|  |  |  |  |  |  | Duration:30min |  |  |  |  |  |  |  |  |  |  |  |  |  |  |  |  |
|  |  |  |  |  |  | freq: 2 times a week |  |  |  |  |  |  |  |  |  |  |  |  |  |  |  |  |
|  |  |  |  |  |  | length of intervention:  12weeks |  |  |  |  |  |  |  |  |  |  |  |  |  |  |  |  |
| Schenkman(Schenkman et al., 2012) | 2012 | Parkinson disease  Hoehn & Yahr1-3 | Group 1:  63.4(11.2)  Group 2:  66.3(10.1) | Group 1:  26(63.4)  Group 2:  24(61.5)  Group 3:  26(63.4) | Group 1:  40(97.6)  Caucasian Group 2:  37(94.9)  Caucasian Group 3:  38(92.7)Caucasian | treadmill | AE program | control | UPDRS-motor;  PDQ39-ADL | -2.5±9.59 | -1.7±9.51 | NA | NA | NA | NA | NA | NA | NA | NA | -1.4±12.91 | -0.5±11.16 | 3 non-injurious falls and 2 soreness or pain |
|  |  |  |  |  |  | Duration:30min |  |  |  |  |  |  |  |  |  |  |  |  |  |  |  |  |
|  |  |  |  |  |  | freq: 3 times a week |  |  |  |  |  |  |  |  |  |  |  |  |  |  |  |  |
|  |  |  |  |  |  | length of intervention:  64weeks |  |  |  |  |  |  |  |  |  |  |  |  |  |  |  |  |
| Frazzitta(Frazzitta et al., 2014) | 2014 | Parkinson disease  Hoehn & Yahr 1-1.5 | Group 1:  67(5)  Group 2:  65(4) | NR | NR | treadmill | control(no therapy) |  | UPDRS-motor | -7.6±3.36 | 0.3±1.28 | NA | NA | NA | NA | NA | NA | NA | NA | NA | NA | NR |
|  |  |  |  |  |  | Duration:30min |  |  |  |  |  |  |  |  |  |  |  |  |  |  |  |  |
|  |  |  |  |  |  | freq: 7 times a week |  |  |  |  |  |  |  |  |  |  |  |  |  |  |  |  |
|  |  |  |  |  |  | length of intervention:  4weeks |  |  |  |  |  |  |  |  |  |  |  |  |  |  |  |  |
| Sage(Sage and Almeida, 2009) | 2009 | Parkinson disease  Hoehn & Yahr1-3 | Group 1:  65.1(9.3)  Group 2:  64.2(10.3)  Group 3:  68.6(8.7) | Group 1:  6(46)  Group 2:  12(67)  Group 3:  7(47) | NR | treadmill | control(sensory attention focused exercise) |  | UPDRS-motor;  TUG | -1.8±7.31 | 1.2±7.81 | -0.7±2.41 | 0±2.33 | NA | NA | NA | NA | NA | NA | NA | NA | NR |
|  |  |  |  |  |  | Duration:20min |  |  |  |  |  |  |  |  |  |  |  |  |  |  |  |  |
|  |  |  |  |  |  | freq: 3 times a week |  |  |  |  |  |  |  |  |  |  |  |  |  |  |  |  |
|  |  |  |  |  |  | length of intervention:  12weeks |  |  |  |  |  |  |  |  |  |  |  |  |  |  |  |  |
| Schenkman(Schenkman et al., 2018) | 2018 | Parkinson disease  Hoehn & Yahr1-2 | Group 1:  64(9)  Group 2:  63(10)  Group 3:  64(10) | Group 1:  22 (43)  Group 2:  27(45)  Group 3:  24 (40) | Group 1:  3(7)  Hispanic  Group 2:  2(4)  Hispanic  Group 3:  1(3) Hispanic | High-intensity treadmill | moderate-intensity treadmill | wait-list control | UPDRS-motor | 0.2±6.3 | 3.2±5.6 | NA | NA | NA | NA | NA | NA | NA | NA | NA | NA | musculoskeletal and connective-tissue disorder |
|  |  |  |  |  |  | Duration:30min |  |  |  |  |  |  |  |  |  |  |  |  |  |  |  |  |
|  |  |  |  |  |  | freq: 4 times a week |  |  |  |  |  |  |  |  |  |  |  |  |  |  |  |  |
|  |  |  |  |  |  | length of intervention:  26weeks |  |  |  |  |  |  |  |  |  |  |  |  |  |  |  |  |
| Nadeau(Nadeau et al., 2014) | 2014 | Parkinson disease  Hoehn & Yahr1-2 | Group 1: 60.1(6.8)  Group 2: 64.3(5.6) | Group 1:  10(91)  Group 2:  9(82) | NR | treadmill training | control |  | MDS-UPDRS  motor;  BDI ;  PDQ39-  cognition;  PDQ39-ADL | -7±10.83 | -4.4±6.41 | NA | NA | NA | NA | -3.1±8.74 | 0.4±7.05 | 0.5±2.15 | -0.1±1.01 | -5.2±13.7 | 0.8±15.08 | no adverse events occurred |
|  |  |  |  |  |  | Duration:60min |  |  |  |  |  |  |  |  |  |  |  |  |  |  |  |  |
|  |  |  |  |  |  | freq: 3 times a week |  |  |  |  |  |  |  |  |  |  |  |  |  |  |  |  |
|  |  |  |  |  |  | length of intervention:  24weeks |  |  |  |  |  |  |  |  |  |  |  |  |  |  |  |  |
| Picelli(Picelli et al., 2016) | 2016 | Parkinson disease  Hoehn & Yahr1-3 | Group 1:  71.2(9.2)  Group 2:  71.6(7.2) | Group 1:  5(56)  Group 2:  4(50) | NR | treadmill | control(no physical treatment) |  | MoCA;  BDI | NA | NA | NA | NA | NA | NA | -5±11.52 | 0±8.86 | 1±5.96 | 1.5±3.83 | NA | NA | NR |
|  |  |  |  |  |  | Duration:45min |  |  |  |  |  |  |  |  |  |  |  |  |  |  |  |  |
|  |  |  |  |  |  | freq: 3 times a week |  |  |  |  |  |  |  |  |  |  |  |  |  |  |  |  |
|  |  |  |  |  |  | length of intervention:  4weeks |  |  |  |  |  |  |  |  |  |  |  |  |  |  |  |  |
| Fatollahkhani(Arfa-Fatollahkhani et al., 2019) | 2019 | Parkinson disease  Hoehn & Yahr1.5-2.5 | Group 1:  60.63(9.4)  Group 2:  61.55(8.6) | Group 1:  7(78)  Group 2:  8(73) | NR | treadmill | control |  | TUG | NA | NA | -3.92±7.64 | -2.56±11.18 | NA | NA | NA | NA | NA | NA | NA | NA | NR |
|  |  |  |  |  |  | Duration:30min |  |  |  |  |  |  |  |  |  |  |  |  |  |  |  |  |
|  |  |  |  |  |  | freq: 2 times a week |  |  |  |  |  |  |  |  |  |  |  |  |  |  |  |  |
|  |  |  |  |  |  | length of intervention:  10weeks |  |  |  |  |  |  |  |  |  |  |  |  |  |  |  |  |
| Canning(Canning et al., 2012) | 2012 | Parkinson disease  Hoehn & Yahr1-2 | Group 1:  60.7(5.9)  Group 2:  62.9(9.9) | Group 1:  5(50)  Group 2:  6(60) | NR | treadmill training | control(usual care) |  | UPDRS-motor; PDQ-39-ADL | 2.4±11.49 | 2.4±7.35 | NA | NA | NA | NA | NA | NA | NA | NA | -5.6±6.26 | 2.5±10.79 | no adverse events |
|  |  |  |  |  |  | Duration:30-40min |  |  |  |  |  |  |  |  |  |  |  |  |  |  |  |  |
|  |  |  |  |  |  | freq: 4 times a week |  |  |  |  |  |  |  |  |  |  |  |  |  |  |  |  |
|  |  |  |  |  |  | length of intervention:6weeks; 12weeks |  |  |  |  |  |  |  |  |  |  |  |  |  |  |  |  |
| Miyai(Miyai et al., 2002) | 2002 | Parkinson disease  Hoehn & Yahr2.5-3 | Group 1:  69.5(1.9)  Group 2:  69.8(1.5) | Group 1:  5(45)  Group 2:  5(56) | NR | treadmill | control(conventional physical therapy) |  | UPDRS-motor;  ADL | -3±4.15 | -1.3±4.2 | NA | NA | NA | NA | NA | NA | NA | NA | -2.9±5.31 | -1.1±3.83 | NR |
|  |  |  |  |  |  | Duration:45min |  |  |  |  |  |  |  |  |  |  |  |  |  |  |  |  |
|  |  |  |  |  |  | freq: 3 times a week |  |  |  |  |  |  |  |  |  |  |  |  |  |  |  |  |
|  |  |  |  |  |  | length of intervention:4weeks; 8weeks |  |  |  |  |  |  |  |  |  |  |  |  |  |  |  |  |
| Ganesan(Ganesan et al., 2015) | 2015 | Parkinson disease  Hoehn & Yahr2-2.5 | All:  58.15(8.7) | NR | NR | treadmill | control(noexercising) |  | UPDRS-motor | -6.85±4.55 | 0.2±3.84 | NA | NA | NA | NA | NA | NA | NA | NA | NA | NA | no adverse events |
|  |  |  |  |  |  | Duration:30min |  |  |  |  |  |  |  |  |  |  |  |  |  |  |  |  |
|  |  |  |  |  |  | freq: 4 times a week |  |  |  |  |  |  |  |  |  |  |  |  |  |  |  |  |
|  |  |  |  |  |  | length of intervention:2weeks; 4weeks |  |  |  |  |  |  |  |  |  |  |  |  |  |  |  |  |
| Shulman(Shulman et al., 2013) | 2013 | Parkinson disease  Hoehn & Yahr1-3 | Group 1:  66.1(9.7)  Group 2:  65.3(11.3) | Group 1:  16(23)  Group 2:  18(22) | NR | treadmill | control(stretching and resistance exercises) |  | UPDRS-motor;  TUG;  BDI | -2.06±7.19 | -3.45±6.1 | -0.5±3.21 | 0.5±6.21 | NA | NA | -0.7±5.88 | 0.7±4.30 | NA | NA | NA | NA | no adverse events |
|  |  |  |  |  |  | higher-intensity: 30min; lower-intensity: 50min |  |  |  |  |  |  |  |  |  |  |  |  |  |  |  |  |
|  |  |  |  |  |  | freq: 3 times a week |  |  |  |  |  |  |  |  |  |  |  |  |  |  |  |  |
|  |  |  |  |  |  | length of intervention: 12weeks |  |  |  |  |  |  |  |  |  |  |  |  |  |  |  |  |
| Picelli(Picelli et al., 2013) | 2013 | Parkinson disease  Hoehn & Yahr1-3 | All:  68.3(8.3) | All:  23(38) | NR | treadmill | Robotic Gait Training | control  (physical therapy) | BBS | NA | NA | NA | NA | 3.6±4.19 | 1±5.11 | NA | NA | NA | NA | NA | NA | no adverse events |
|  |  |  |  |  |  | Duration:45min |  |  |  |  |  |  |  |  |  |  |  |  |  |  |  |  |
|  |  |  |  |  |  | freq: 3 times a week |  |  |  |  |  |  |  |  |  |  |  |  |  |  |  |  |
|  |  |  |  |  |  | length of intervention:4weeks; 16weeks |  |  |  |  |  |  |  |  |  |  |  |  |  |  |  |  |
| Fisher(Fisher et al., 2008) | 2008 | Parkinson disease  Hoehn & Yahr1-2 | Group 1:  64.0(14.5)  Group 2:  63.1(11.5) | Group 1:  6(60)  Group 2:  8(80) | NR | treadmill | control(zero-intensity education) |  | UPDRS-motor;  UPDRS-mental；  ADL | -3.8±8.17 | -2.7±8.15 | NA | NA | NA | NA | NA | NA | 0±1.97 | -0.3±0.91 | -1.5±2.81 | -0.9±3.90 | no adverse events |
|  |  |  |  |  |  | Duration:45min |  |  |  |  |  |  |  |  |  |  |  |  |  |  |  |  |
|  |  |  |  |  |  | freq: 3 times a week |  |  |  |  |  |  |  |  |  |  |  |  |  |  |  |  |
|  |  |  |  |  |  | length of intervention: 8weeks |  |  |  |  |  |  |  |  |  |  |  |  |  |  |  |  |
| Khalil(Khalil et al., 2017) | 2017 | Parkinson disease  Hoehn & Yahr1-4 | Group 1:  58.4(13.5)  Group 2:  60.7(15.4) | Group 1:  12(75)  Group 2:  7(100) | NR | walking | control(standard care) |  | MDS-UPDRS-  motor | -11.2±17.43 | 4.1±18.27 | NA | NA | NA | NA | NA | NA | NA | NA | NA | NA | no serious adverse events ; neck pain or low back pain and knee pain |
|  |  |  |  |  |  | Duration:45min |  |  |  |  |  |  |  |  |  |  |  |  |  |  |  |  |
|  |  |  |  |  |  | freq: 1 times a week |  |  |  |  |  |  |  |  |  |  |  |  |  |  |  |  |
|  |  |  |  |  |  | length of intervention: 8weeks |  |  |  |  |  |  |  |  |  |  |  |  |  |  |  |  |
| Beck(Beck et al., 2018) | 2018 | Parkinson disease  Hoehn & Yahr1-4 | Group 1:  68.63(9.9)  Group 2:  71.27(6.6) | Group 1:  15(79)  Group 2:  10(91) | NR | walking | control |  | UPDRS-motor; MoCA | -5.44±10.51 | 0.05±9.39 | NA | NA | NA | NA | NA | NA | 0.37±5.21 | 0.7±6.26 | NA | NA | no adverse effects |
|  |  |  |  |  |  | Duration:60 minutes |  |  |  |  |  |  |  |  |  |  |  |  |  |  |  |  |
|  |  |  |  |  |  | freq: 3 times a week |  |  |  |  |  |  |  |  |  |  |  |  |  |  |  |  |
|  |  |  |  |  |  | length of intervention: 12weeks |  |  |  |  |  |  |  |  |  |  |  |  |  |  |  |  |
| Reuter(Reuter et al., 2011) | 2011 | Parkinson disease  Hoehn & Yahr2-3 | Group 1:  63(3.1)  Group 2:  62.1(2.5) | Group 1:  45(50)  Group 2:  45(50) | NR | Nordic walking;  Walking | control(Flexibility and relaxation) |  | UPDRS-motor | -6.03±8.23 | 0.14±8.20 | NA | NA | NA | NA | NA | NA | NA | NA | NA | NA | one hypotension after walking uphill in hot weather |
|  |  |  |  |  |  | Duration:70min |  |  |  |  |  |  |  |  |  |  |  |  |  |  |  |  |
|  |  |  |  |  |  | freq: 3 times a week |  |  |  |  |  |  |  |  |  |  |  |  |  |  |  |  |
|  |  |  |  |  |  | length of intervention: 24Weeks |  |  |  |  |  |  |  |  |  |  |  |  |  |  |  |  |
| Cugusia(Cugusi et al., 2015) | 2015 | Parkinson disease  Hoehn & Yahr1-3 | Group 1:  68.1(8.7)  Group 2:  66.6(7.3) | Group 1:  8(80)  Group 2:  8(80) | NR | walking | Control |  | UPDRS-motor; TUG;  BBS;  BDI | -6.5±11.75 | 1±11.85 | -0.7±2.48 | 0.9±2.3 | 6.6±6.85 | -3±7.39 | -5.2±8.19 | 0.4±12.05 | NA | NA | NA | NA | no adverse events |
|  |  |  |  |  |  | Duration:60 minutes |  |  |  |  |  |  |  |  |  |  |  |  |  |  |  |  |
|  |  |  |  |  |  | freq: 2 times a week |  |  |  |  |  |  |  |  |  |  |  |  |  |  |  |  |
|  |  |  |  |  |  | length of intervention: 12weeks |  |  |  |  |  |  |  |  |  |  |  |  |  |  |  |  |
| Bang(Bang and Shin, 2017) | 2017 | Parkinson disease  Hoehn & Yahr1-3 | Group 1:  58.30(7.7)  Group 2:  60.60(6.7) | Group 1:  5(50)  Group 2:  4(40) | NR | walking | treadmill |  | UPDRS-motor; TUG;  BBS | -6.3±2.54 | -3.7±2.71 | -4.72±2.0 | -3.48±1.95 | 5.9±3.94 | 2.7±4.23 | NA | NA | NA | NA | NA | NA | NR |
|  |  |  |  |  |  | Duration:60 minutes |  |  |  |  |  |  |  |  |  |  |  |  |  |  |  |  |
|  |  |  |  |  |  | freq: 5 times a week |  |  |  |  |  |  |  |  |  |  |  |  |  |  |  |  |
|  |  |  |  |  |  | length of intervention: 4weeks |  |  |  |  |  |  |  |  |  |  |  |  |  |  |  |  |
| Cheng(Cheng et al., 2017) | 2017 | Parkinson disease  Hoehn & Yahr1-3 | Group 1:  65.8(11.5)  Group 2:  67.3(6.4) | Group 1:  9(75)  Group 2:  8(67) | NR | walking | control exercise |  | UPDRS-motor; TUG | -1.6±4.10 | 0.5±6.20 | -1.4±4.17 | 0.2±2.75 | NA | NA | NA | NA | NA | NA | NA | NA | muscle soreness, no other adverse effects such as falls were reported |
|  |  |  |  |  |  | Duration:30 minutes |  |  |  |  |  |  |  |  |  |  |  |  |  |  |  |  |
|  |  |  |  |  |  | freq: 12 times in 4-6week |  |  |  |  |  |  |  |  |  |  |  |  |  |  |  |  |
|  |  |  |  |  |  | length of intervention:  4weeks; 8weeks |  |  |  |  |  |  |  |  |  |  |  |  |  |  |  |  |
| Bello(Bello et al., 2013) | 2013 | Parkinson disease  Hoehn & Yahr1-3 | Group 1:  58.0(9.4)  Group 2:  59.5(11.3) | Group 1:  7(64)  Group 2:  6(55) | NR | walking | treadmill |  | UPDRS-motor; TUG | -3.89±2.53 | 2.63±2.57 | 0.13±0.60 | -1.61±1.51 | NA | NA | NA | NA | NA | NA | NA | NA | NR |
|  |  |  |  |  |  | Duration:no report |  |  |  |  |  |  |  |  |  |  |  |  |  |  |  |  |
|  |  |  |  |  |  | freq: 3 times a week |  |  |  |  |  |  |  |  |  |  |  |  |  |  |  |  |
|  |  |  |  |  |  | length of intervention:  5weeks |  |  |  |  |  |  |  |  |  |  |  |  |  |  |  |  |
| Song(Song et al., 2018) | 2018 | Parkinson disease  Hoehn & Yahr1-3 | Group 1:  68(7)  Group 2:  65(7) | Group 1:  15(48)  Group 2:  9(31) | NR | dance | control |  | TUG;  MoCA | 0.15±2.27 | -0.49±2.05 | NA | NA | NA | NA | NA | NA | 0.9±2.79 | 0.2±2.52 | NA | NA | no adverse events occurred |
|  |  |  |  |  |  | Duration:15min |  |  |  |  |  |  |  |  |  |  |  |  |  |  |  |  |
|  |  |  |  |  |  | freq: 3 times a week |  |  |  |  |  |  |  |  |  |  |  |  |  |  |  |  |
|  |  |  |  |  |  | length of intervention: 12weeks |  |  |  |  |  |  |  |  |  |  |  |  |  |  |  |  |
| Michels(Michels et al., 2018) | 2018 | Parkinson disease  Hoehn & Yahr2-2.5 | All:  69.2(8.7) | All:  6(46) | Most of whom were white | dance | control(support group) |  | MDS-UPDRS-  motor;  TUG;  MoCA;  BBS;  BDI | -4.12±11.12 | -1.79±10.71 | -0.5±1.60 | -0.31±9.58 | 2.55±2.31 | 6.25±10.33 | 1.33±6.18 | 1±2.07 | 0.44±2.30 | -0.5±1.32 | NA | NA | no serious adverse events occur. low back pain |
|  |  |  |  |  |  | Duration:60 minutes |  |  |  |  |  |  |  |  |  |  |  |  |  |  |  |  |
|  |  |  |  |  |  | freq: 1 time a week |  |  |  |  |  |  |  |  |  |  |  |  |  |  |  |  |
|  |  |  |  |  |  | length of intervention: 10weeks |  |  |  |  |  |  |  |  |  |  |  |  |  |  |  |  |
| Volpe1(Volpe et al., 2013) | 2013 | Parkinson disease  Hoehn & Yahr1-2.5 | Group 1:  61.6(4.5)  Group 2:  65.0(5.3) | Group 1:  7(58)  Group 2:  6(50) | NR | dance | control |  | UPDRS-motor;  PDQ-39-ADL;  BBS | -8.06±3.86 | -2.92±3.31 | NA | NA | 10±8.25 | 4.84±9.58 | NA | NA | NA | NA | -8.44±11.24 | -4.97±7.63 | a non-injurious fall |
|  |  |  |  |  |  | Duration:90 minutes |  |  |  |  |  |  |  |  |  |  |  |  |  |  |  |  |
|  |  |  |  |  |  | freq: 1 time a week |  |  |  |  |  |  |  |  |  |  |  |  |  |  |  |  |
|  |  |  |  |  |  | length of intervention:  24weeks |  |  |  |  |  |  |  |  |  |  |  |  |  |  |  |  |
| Shanahan(Shanahan et al., 2017) | 2017 | Parkinson disease  Hoehn & Yahr1-2.5 | Group 1:  69(10)  Group 2:  69(8) | Group 1:  13(65)  Group 2:  13(62) | NR | dance | control(usual care) |  | UPDRS-motor; PDQ39-ADL | -0.5±6.75 | 6.55±13.79 | NA | NA | NA | NA | NA | NA | NA | NA | -4.8±12.54 | -2.15±13.31 | no adverse effects were reported |
|  |  |  |  |  |  | Duration:20 minutes |  |  |  |  |  |  |  |  |  |  |  |  |  |  |  |  |
|  |  |  |  |  |  | freq: 3 times a week |  |  |  |  |  |  |  |  |  |  |  |  |  |  |  |  |
|  |  |  |  |  |  | length of intervention:  10weeks |  |  |  |  |  |  |  |  |  |  |  |  |  |  |  |  |
| Hackney(Hackney and Earhart, 2009) | 2009 | Parkinson disease  Hoehn & Yahr1-3 | Group 1:  68.2(1.4)  Group 2:  66.5(2.8) | Group 1:  11(79)  Group 2:  12(71) | NR | dance | Control(no intervention) |  | UPDRS-motor; TUG;  BBS | -2.6±12.58 | 5±10.33 | -2.1±4.86 | 2±9.28 | 4±4.95 | -1.2±9.32 | NA | NA | NA | NA | NA | NA | NR |
|  |  |  |  |  |  | Duration:60 minutes |  |  |  |  |  |  |  |  |  |  |  |  |  |  |  |  |
|  |  |  |  |  |  | freq: 2 times a week |  |  |  |  |  |  |  |  |  |  |  |  |  |  |  |  |
|  |  |  |  |  |  | length of intervention:  13weeks |  |  |  |  |  |  |  |  |  |  |  |  |  |  |  |  |
| Rawson(Rawson et al., 2019) | 2019 | Parkinson disease  Hoehn & Yahr1-4 | All:  67.2(8.9) | All:  56(58) | NR | tango | treadmill | control(stretching) | MDS-UPDRS-  motor;  PDQ39-ADL | -1.95±11.50 | -2.65±11.54 | NA | NA | NA | NA | NA | NA | NA | NA | 0.36±1.40 | 0.27±2.06 | no adverse events were reported |
|  |  |  |  |  |  | Duration:60 minutes |  |  |  |  |  |  |  |  |  |  |  |  |  |  |  |  |
|  |  |  |  |  |  | freq: 2 times a week |  |  |  |  |  |  |  |  |  |  |  |  |  |  |  |  |
|  |  |  |  |  |  | length of intervention:12weeks; 24weeks |  |  |  |  |  |  |  |  |  |  |  |  |  |  |  |  |
| Kalyania(Kalyani et al., 2019) | 2019 | Parkinson disease  Hoehn & Yahr1-3 | Group 1:  65.2(11.9)  Group 2:  66.5(7.7) | Group 1:  3(17.6)  Group 2:  10(62.5) | NR | dance | control |  | HADS;  PDQ39-  Cognition;  PDQ39-ADL | NA | NA | NA | NA | NA | NA | -1.53±2.57 | 1.87±3.15 | 5.88±20.30 | -2.34±14.18 | -1.47±7.17 | 1.75±6.98 | no adverse events were reported |
|  |  |  |  |  |  | Duration:60 minutes |  |  |  |  |  |  |  |  |  |  |  |  |  |  |  |  |
|  |  |  |  |  |  | freq: 2 times a week |  |  |  |  |  |  |  |  |  |  |  |  |  |  |  |  |
|  |  |  |  |  |  | length of intervention:  12weeks |  |  |  |  |  |  |  |  |  |  |  |  |  |  |  |  |
| Duncan(Duncan and Earhart, 2012) | 2012 | Parkinson disease  Hoehn & Yahr1-4 | Group 1:  69.3(1.9)  Group2:  69.0(1.5) | Group 1:  15(58)  Group 2:  15(58) | NR | tango | control(no intervention) |  | MDS-UPDRS-  motor | -10.3±11.48 | -2.8±9.45 | NA | NA | NA | NA | NA | NA | NA | NA | NA | NA | NR |
|  |  |  |  |  |  | Duration:60 minutes |  |  |  |  |  |  |  |  |  |  |  |  |  |  |  |  |
|  |  |  |  |  |  | freq: 2 times a week |  |  |  |  |  |  |  |  |  |  |  |  |  |  |  |  |
|  |  |  |  |  |  | length of intervention:12weeks; 24weeks; 48weeks |  |  |  |  |  |  |  |  |  |  |  |  |  |  |  |  |
| Solla(Solla et al., 2019) | 2019 | Parkinson disease  Hoehn & Yahr1-3 | Group 1:  67.8(5.9)  Group 2:  67.1(6.3) | Group 1:  6(60)  Group 2:  7(70) | NR | Sardinian folk dance | control(usual care) |  | UPDRS-motor; TUG;  BBS | -5.3±6.98 | 0.88±6.67 | -1.8±0.9 | -0.48±1.19 | 6.9±3.55 | -0.7±5.64 | NA | NA | NA | NA | NA | NA | no adverse events were reported |
|  |  |  |  |  |  | Duration:90 minutes |  |  |  |  |  |  |  |  |  |  |  |  |  |  |  |  |
|  |  |  |  |  |  | freq: 2 times a week |  |  |  |  |  |  |  |  |  |  |  |  |  |  |  |  |
|  |  |  |  |  |  | length of intervention:  12weeks |  |  |  |  |  |  |  |  |  |  |  |  |  |  |  |  |
| Romenets(Rios Romenets et al., 2015) | 2015 | Parkinson disease  Hoehn & Yahr1-3 | Group 1:  63.2(9.9)  Group 2:  64.3(8.1) | Group 1:  12(67)  Group 2:  7(47) | NR | dance | control(self-directed exercise） |  | UPDRS-moto; TUG;  BDI;  MoCA | -1.6±10.15 | -1.2±14.03 | -1.3±1.80 | 0.1±2.36 | NA | NA | -0.2±6.91 | -0.4±4.91 | 0.4±2.26 | -0.6±3.02 | NA | NA | falls, mild fatigue and muscle cramps |
|  |  |  |  |  |  | Duration:60 minutes |  |  |  |  |  |  |  |  |  |  |  |  |  |  |  |  |
|  |  |  |  |  |  | freq: 2 times a week |  |  |  |  |  |  |  |  |  |  |  |  |  |  |  |  |
|  |  |  |  |  |  | length of intervention:  12weeks |  |  |  |  |  |  |  |  |  |  |  |  |  |  |  |  |
| Kolk(van der Kolk et al., 2019) | 2019 | Parkinson disease  Hoehn & Yahr1-2 | Group 1:  59.3(8.3)  Group 2:  59.4(9.3) | Group 1:  42(65)  Group 2:  38(58) | NR | cycling | control(stretching） |  | UPDRS-motor; BBS;  HADS;  PDQ39-ADL | -0.5±2.61 | 4.2±2.61 | NA | NA | 0.1±0.6 | 0.3±0.6 | 0.3±0.56 | -0.2±0.6 | -0.6±0.46 | -0.4±0.46 | 1.1±2.25 | 2.3±2.25 | arthralgia or back pain |
|  |  |  |  |  |  | Duration:33min |  |  |  |  |  |  |  |  |  |  |  |  |  |  |  |  |
|  |  |  |  |  |  | freq: 2.6 times a week |  |  |  |  |  |  |  |  |  |  |  |  |  |  |  |  |
|  |  |  |  |  |  | length of intervention:  24weeks |  |  |  |  |  |  |  |  |  |  |  |  |  |  |  |  |
| Sacheli(Sacheli et al., 2019) | 2019 | Parkinson disease  Hoehn & Yahr1-3 | Group 1:  66.76(5.9)  Group 2:  67.85(8.5) | Group 1:  13(65)  Group 2:  9(60) | NR | cycling | stretching control |  | MDS-UPDRS-  motor;  HADS;  MoCA | 0.65±10.99 | -1.69±13.76 | NA | NA | NA | NA | -0.09±6.63 | -0.77±7.59 | -0.41±1.96 | -0.69±2.23 | NA | NA | no adverse effects |
|  |  |  |  |  |  | Duration:30-50min |  |  |  |  |  |  |  |  |  |  |  |  |  |  |  |  |
|  |  |  |  |  |  | freq: 3 times a week |  |  |  |  |  |  |  |  |  |  |  |  |  |  |  |  |
|  |  |  |  |  |  | length of intervention:  12weeks |  |  |  |  |  |  |  |  |  |  |  |  |  |  |  |  |
| Ridgel(Ridgel and Ault, 2019) | 2019 | Parkinson disease  Hoehn & Yahr1-3 | Group 1:  69.9(7.4)  Group 2:  70.0(6.4) | Group 1:  4(50)  Group 2:  5(63) | NR | cycling | control(stretching) |  | UPDRS-motor; TUG | -4.26±1.95 | 0.77±3.94 | -2.14±2.74 | -0.11±2.64 | NA | NA | NA | NA | NA | NA | NA | NA | NR |
|  |  |  |  |  |  | Duration:40 minutes |  |  |  |  |  |  |  |  |  |  |  |  |  |  |  |  |
|  |  |  |  |  |  | freq: 3-4 times a week |  |  |  |  |  |  |  |  |  |  |  |  |  |  |  |  |
|  |  |  |  |  |  | length of intervention:  15days |  |  |  |  |  |  |  |  |  |  |  |  |  |  |  |  |
| Arcolin(Arcolin et al., 2016) | 2015 | Parkinson disease  Hoehn & Yahr1.5-3 | Group 1:  68.7(8.3)  Group 2:  67.8(8.8) | Group 1:  9(56)  Group 2:  6(46) | NR | cycling | treadmill training |  | UPDRS-motor; TUG | -6.3±10.55 | -6±9.36 | -0.69±0.46 | -1.63±0.40 | NA | NA | NA | NA | NA | NA | NA | NA | NR |
|  |  |  |  |  |  | Duration:60 minutes |  |  |  |  |  |  |  |  |  |  |  |  |  |  |  |  |
|  |  |  |  |  |  | freq: 5 times a week |  |  |  |  |  |  |  |  |  |  |  |  |  |  |  |  |
|  |  |  |  |  |  | length of intervention:  3weeks |  |  |  |  |  |  |  |  |  |  |  |  |  |  |  |  |
| Tollar(Tollár et al., 2019) | 2019 | Parkinson disease  Hoehn & Yahr2-3 | Group 1:  70.6(4.10)  Group 2:  70.0(4.69)  Group 3:  67.5(4.28) | Group 1:  12(48)  Group 2:  11(44)  Group 3:  13(54) | NR | cycling | exergaming exercise | wait-listed control | BBS | NA | NA | NA | NA | 4.2±4.17 | -1.4±5.91 | NA | NA | NA | NA | NA | NA | no adverse events |
|  |  |  |  |  |  | Duration:45 minutes |  |  |  |  |  |  |  |  |  |  |  |  |  |  |  |  |
|  |  |  |  |  |  | freq: 5 times in a weeks |  |  |  |  |  |  |  |  |  |  |  |  |  |  |  |  |
|  |  |  |  |  |  | length of intervention:  5weeks |  |  |  |  |  |  |  |  |  |  |  |  |  |  |  |  |
| Sharma(Sharma et al., 2015) | 2015 | Parkinson disease  Hoehn & Yahr1-2 | Group 1:  62.8(13.2)  Group 2:  73.4(6.5) | Group 1:  2(25)  Group 2:  5(80) | NR | yoga | control |  | UPDRS-motor | -8.29±4.44 | 1.7±5.49 | NA | NA | NA | NA | NA | NA | NA | NA | NA | NA | NR |
|  |  |  |  |  |  | Duration:60 minutes |  |  |  |  |  |  |  |  |  |  |  |  |  |  |  |  |
|  |  |  |  |  |  | freq: 2 times a week |  |  |  |  |  |  |  |  |  |  |  |  |  |  |  |  |
|  |  |  |  |  |  | length of intervention: 6weeks; 12weeks |  |  |  |  |  |  |  |  |  |  |  |  |  |  |  |  |
| Ni(Ni et al., 2016b) | 2016 | Parkinson disease  Hoehn & Yahr1-3 | All:  72.2(6.5) | Group 1:  11(85)  Group 2:  9(64) | NR | yoga | non-exercise control |  | UPDRS-motor; TUG;  BBS | -10.9±5.13 | 0.4±2.52 | -2.3±2.9 | 0.3±0.84 | 4.2±2.3 | 0.4±0.7 | NA | NA | NA | NA | NA | NA | no adverse events |
|  |  |  |  |  |  | Duration:60min |  |  |  |  |  |  |  |  |  |  |  |  |  |  |  |  |
|  |  |  |  |  |  | freq: 2 times a week |  |  |  |  |  |  |  |  |  |  |  |  |  |  |  |  |
|  |  |  |  |  |  | length of intervention:  12weeks |  |  |  |  |  |  |  |  |  |  |  |  |  |  |  |  |
| Ni(Ni et al., 2016a) | 2016 | Parkinson disease  Hoehn & Yahr1-3 | Group 1:  71.2(6.5)  Group 2:  74.9(8.3) | Group 1:  11(73)  Group 2:  6(100) | NR | yoga | non-exercise control |  | PDQ-39-ADL;  cognition | NA | NA | NA | NA | NA | NA | NA | NA | 5±1.41 | -0.1±1.33 | -1.4±3.48 | 1.2±1.26 | no adverse events |
|  |  |  |  |  |  | Duration:60 minutes |  |  |  |  |  |  |  |  |  |  |  |  |  |  |  |  |
|  |  |  |  |  |  | freq: 2 times a week |  |  |  |  |  |  |  |  |  |  |  |  |  |  |  |  |
|  |  |  |  |  |  | length of intervention:  12weeks |  |  |  |  |  |  |  |  |  |  |  |  |  |  |  |  |
| Khuzema(Khuzema et al., 2020) | 2020 | Parkinson disease  Hoehn & Yahr2.5-3 | Group 1:  68.11(4.2)  Group 2:  72(5.2)  Group 3:  70.89(6.0) | Group 1:  6(67)  Group 2:  6(67)  Group 3:  7(78) | NR | yoga | taichi | control(re-gular  balance exercises) | TUG; BBS | NA | NA | -1.39±13.36 | -1.38±6.87 | 3.78±4.74 | 6.33±8.54 | NA | NA | NA | NA | NA | NA | no adverse events |
|  |  |  |  |  |  | Duration:30-40min |  |  |  |  |  |  |  |  |  |  |  |  |  |  |  |  |
|  |  |  |  |  |  | freq: 5 times a week |  |  |  |  |  |  |  |  |  |  |  |  |  |  |  |  |
|  |  |  |  |  |  | length of intervention:  8weeks |  |  |  |  |  |  |  |  |  |  |  |  |  |  |  |  |
| Kwok(Kwok et al., 2019) | 2019 | Parkinson disease  Hoehn & Yahr1-3 | All:  63.6(8.7) | All:  65 (47.1) | NR | yoga | control(re-sistance training exercise) |  | MDS-UPDRS-  motor;  TUG;  HADS;  ADL | -12.4±13.46 | -8.39±14.41 | -5.18±13.90 | -0.58±14.39 | NA | NA | -3.16±3.13 | -0.16±3.68 | NA | NA | -2.22±4.86 | 0.45±5.16 | mild knee pain, no serious adverse events were reported |
|  |  |  |  |  |  | Duration:90 minutes |  |  |  |  |  |  |  |  |  |  |  |  |  |  |  |  |
|  |  |  |  |  |  | freq: 1 time a week |  |  |  |  |  |  |  |  |  |  |  |  |  |  |  |  |
|  |  |  |  |  |  | length of intervention:8weeks; 20weeks |  |  |  |  |  |  |  |  |  |  |  |  |  |  |  |  |
| Cheung(Cheung et al., 2018) | 2018 | Parkinson disease  Hoehn & Yahr1-3 | Group 1:  63.5(8.5)  Group 2:  65.8(6.6) | NR | Group 1:  5(50)  white Group 2:  5(50)  white | yoga | wait-list control |  | UPDRS-motor; MoCA;  BDI | -8.6±6.28 | -1.9±6.64 | NA | NA | NA | NA | 0.1±5.14 | 1.5±4.52 | 1.2±1.91 | 1.4±2.08 | NA | NA | no adverse events |
|  |  |  |  |  |  | Duration:60 minutes |  |  |  |  |  |  |  |  |  |  |  |  |  |  |  |  |
|  |  |  |  |  |  | freq: 2 times a week |  |  |  |  |  |  |  |  |  |  |  |  |  |  |  |  |
|  |  |  |  |  |  | length of intervention:12weeks |  |  |  |  |  |  |  |  |  |  |  |  |  |  |  |  |
| Marieke(Van Puymbroeck et al., 2018) | 2018 | Parkinson disease  Hoehn & Yahr1.5-3 | Group 1:  65.53(6.1)  Group 2:  70.5(4.4) | Group 1:  10(66.7)  Group 2:  7(58.3) | Group 1:  15(100)  white  Group 2:  12(100) white | yoga | wait-list control |  | MDS-UPDRS-  motor | -6.4±15.03 | -1.16±11.65 | NA | NA | NA | NA | NA | NA | NA | NA | NA | NA | NR |
|  |  |  |  |  |  | Duration:60min |  |  |  |  |  |  |  |  |  |  |  |  |  |  |  |  |
|  |  |  |  |  |  | freq: 2 times a week |  |  |  |  |  |  |  |  |  |  |  |  |  |  |  |  |
|  |  |  |  |  |  | length of intervention:8weeks |  |  |  |  |  |  |  |  |  |  |  |  |  |  |  |  |
| Poier(Poier et al., 2019) | 2019 | Parkinson disease  Hoehn & Yahr1-3 | Group 1:  68.50(8.1)  Group 2:  68.87(10.9) | Group 1:  9(64.2)  Group 2:  3(2) | NR | Tai Chi | dance |  | PDQ39-ADL | NA | NA | NA | NA | NA | NA | NA | NA | NA | NA | 7.45±22.60 | -1.11±25.50 | one fall at home |
|  |  |  |  |  |  | Duration:60 minutes |  |  |  |  |  |  |  |  |  |  |  |  |  |  |  |  |
|  |  |  |  |  |  | freq: 1 time a week |  |  |  |  |  |  |  |  |  |  |  |  |  |  |  |  |
|  |  |  |  |  |  | length of intervention:  10weeks |  |  |  |  |  |  |  |  |  |  |  |  |  |  |  |  |
| Gao(Gao et al., 2014) | 2014 | Parkinson disease  Hoehn & Yahr1-4 | Group 1:  69.54(7.3)  Group 2:  68.28(8.5) | Group 1:  23(62.2)  Group 2:  27(69.2) | NR | Tai Chi | control(no intervention) |  | UPDRS-motor; TUG;  BBS | -8.05±10.91 | -1.9±11.25 | -1.37±2.93 | 0.03±2.85 | 4.16±9.62 | 0.39±9.15 | NA | NA | NA | NA | NA | NA | NR |
|  |  |  |  |  |  | Duration:60 minutes |  |  |  |  |  |  |  |  |  |  |  |  |  |  |  |  |
|  |  |  |  |  |  | freq: 3 times a week |  |  |  |  |  |  |  |  |  |  |  |  |  |  |  |  |
|  |  |  |  |  |  | length of intervention:  12weeks |  |  |  |  |  |  |  |  |  |  |  |  |  |  |  |  |
| Choi(Choi, 2016) | 2016 | Parkinson disease  Hoehn & Yahr1-2 | Group 1:  60.81(7.6)  Group 2:  65.54(6.8) | NR | NR | Tai Chi | control |  | TUG;  ADL | NA | NA | -0.36±0.90 | 1.26±3.70 | NA | NA | NA | NA | NA | NA | -2.09±2.86 | 0.33±3.55 | NR |
|  |  |  |  |  |  | Duration:30 minutes |  |  |  |  |  |  |  |  |  |  |  |  |  |  |  |  |
|  |  |  |  |  |  | freq: 1 time a week |  |  |  |  |  |  |  |  |  |  |  |  |  |  |  |  |
|  |  |  |  |  |  | length of intervention:  12weeks |  |  |  |  |  |  |  |  |  |  |  |  |  |  |  |  |
| Li(Li et al., 2012) | 2012 | Parkinson disease  Hoehn & Yahr1-4 | Group 1:  68(9)  Group 2:  69(8)  Group 3:  69(9) | Group 1:  45(69.2)  Group 2:  38(58.5)  Group 3:  39(60) | NR | Tai Chi | control(re-sistance training) | control(str-etching) | UPDRS-motor; TUG | -6.42±5.02 | -1.4±6.96 | -1.05±2.80 | -0.02±3.32 | NA | NA | NA | NA | NA | NA | NA | NA | no major adverse events were noted |
|  |  |  |  |  |  | Duration:60 minutes |  |  |  |  |  |  |  |  |  |  |  |  |  |  |  |  |
|  |  |  |  |  |  | freq: 2 times a week |  |  |  |  |  |  |  |  |  |  |  |  |  |  |  |  |
|  |  |  |  |  |  | length of intervention:  24weeks |  |  |  |  |  |  |  |  |  |  |  |  |  |  |  |  |
| Vergara-Diaz(Vergara-Diaz et al., 2018) | 2018 | Parkinson disease  Hoehn & Yahr1-2.5 | Group 1:  65.7(3.86)  Group 2:  62(7.77) | Group 1:  9(56.3)  Group 2:  7(43.8) | Group 1:  16(100)  Non-Hispanic Group 2:  14(87.5)  Non-Hispanic | Tai Chi | usual care |  | UPDRS-motor; TUG;  PDQ39-ADL | 0.62±9.02 | 4.68±9.35 | -0.58±2.62 | 0±1.78 | NA | NA | NA | NA | NA | NA | -4.91±11.10 | -2.14±11.40 | no serious adverse event. back pain, falls/injuries, illness, and pain |
|  |  |  |  |  |  | Duration:60 minutes |  |  |  |  |  |  |  |  |  |  |  |  |  |  |  |  |
|  |  |  |  |  |  | freq: 2 times a week |  |  |  |  |  |  |  |  |  |  |  |  |  |  |  |  |
|  |  |  |  |  |  | length of intervention:12weeks; 24weeks |  |  |  |  |  |  |  |  |  |  |  |  |  |  |  |  |
| Hackney(Hackney and Earhart, 2008) | 2008 | Parkinson disease  Hoehn & Yahr1.5-3 | Group 1:  64.9(8.3)  Group 2:  62.6(10.2) | Group 1:  11(84.6)  Group 2:  10(76.9) | NR | Tai Chi | control |  | UPDRS-motor; TUG;  BBS | -1.5±6.6 | 4.3±5.6 | -1±0.1 | -0.1±1.1 | 3.3±3 | -0.5±2.1 | NA | NA | NA | NA | NA | NA | NR |
|  |  |  |  |  |  | Duration:60 minutes |  |  |  |  |  |  |  |  |  |  |  |  |  |  |  |  |
|  |  |  |  |  |  | freq: 2 times a week |  |  |  |  |  |  |  |  |  |  |  |  |  |  |  |  |
|  |  |  |  |  |  | length of intervention:  12weeks |  |  |  |  |  |  |  |  |  |  |  |  |  |  |  |  |
| Amano(Amano et al., 2013) | 2013 | Parkinson disease  Hoehn & Yahr2-3 | Group 1:  66(11)  Group 2:  66(7) | Group 1:  7(46.7)  Group 2:  7(77.8) | NR | Tai Chi | control |  | UPDRS-motor | 0.3±5.47 | -1.1±5.25 | NA | NA | NA | NA | NA | NA | NA | NA | NA | NA | NR |
|  |  |  |  |  |  | Duration:60 minutes |  |  |  |  |  |  |  |  |  |  |  |  |  |  |  |  |
|  |  |  |  |  |  | freq: 2 times a week |  |  |  |  |  |  |  |  |  |  |  |  |  |  |  |  |
|  |  |  |  |  |  | length of intervention:  16weeks |  |  |  |  |  |  |  |  |  |  |  |  |  |  |  |  |
| Choi(Choi et al., 2013) | 2013 | Parkinson disease  Hoehn & Yahr1-2 | Group 1:  60.81(7.6)  Group 2:  65.54(6.8) | NR | NR | Tai Chi | control |  | MDS-UPDRS-  motor;  TUG | -6.72±8.81 | -1.23±8.68 | -0.36±0.90 | 1.26±3.70 | NA | NA | NA | NA | NA | NA | NA | NA | NR |
|  |  |  |  |  |  | Duration:60 minutes |  |  |  |  |  |  |  |  |  |  |  |  |  |  |  |  |
|  |  |  |  |  |  | freq: 2 times a week |  |  |  |  |  |  |  |  |  |  |  |  |  |  |  |  |
|  |  |  |  |  |  | length of intervention:  12weeks |  |  |  |  |  |  |  |  |  |  |  |  |  |  |  |  |

Abbreviations: NA=not applicable or not available; NR=not reported.

# Supplementary Table 2. Assessment of inconsistency

**Supplementary Table 2a: Design inconsistency**

|  | **Chi-square** | ***P* value for test of global inconsistency** |
| --- | --- | --- |
| UPDRS-motor | 2.93 | 0.570 |
| TUG | 2.34 | 0.674 |
| BBS | 0.95 | 0.622 |
| ADL | 6.47 | 0.091 |

**Supplementary Table 2b: side-split methods**

**UPDRS-motor:**

| **Side** | **Direct Coef.** | **Std. Err.** | **Indirect**  **Coef.** | **Std. Err.** | **Difference**  **Coef.** | **Std. Err.** | ***P*>z** |
| --- | --- | --- | --- | --- | --- | --- | --- |
| AB | -4.593 | 1.699 | -1.792 | 1.985 | -2.801 | 2.614 | 0.284 |
| AC | -0.300 | 4.315 | -0.886 | 1.851 | 0.586 | 4.696 | 0.901 |
| AD | 0.853 | 3.542 | -2.267 | 1.701 | 3.119 | 3.936 | 0.428 |
| AG | 3.309 | 0.933 | 2.381 | 2.094 | 0.928 | 2.304 | 0.687 |
| BG | 5.287 | 1.763 | 8.088 | 1.918 | -2.802 | 2.614 | 0.284 |
| CG | 4.021 | 1.648 | 3.439 | 4.399 | 0.582 | 4.694 | 0.901 |
| DG | 4.870 | 1.362 | 4.095 | 6.522 | 0.775 | 6.677 | 0.908 |
| EG | 8.077 | 1.558 | 4.819 | 169.138 | 3.259 | 169.145 | 0.985 |
| FG | 4.148 | 1.319 | 6.150 | 180.953 | -2.002 | 180.959 | 0.991 |
| AB | -4.593 | 1.699 | -1.792 | 1.985 | -2.801 | 2.614 | 0.284 |

**TUG:**

| **Side** | **Direct**  **Coef.** | **Std. Err.** | **Indirect**  **Coef.** | **Std. Err.** | **Difference**  **Coef.** | **Std. Err.** | ***P*>\|z\|** |
| --- | --- | --- | --- | --- | --- | --- | --- |
| A B | 0.665 | 0.691 | -0.287 | 1.270 | 0.952 | 1.446 | 0.51 |
| A C | 0.940 | 0.712 | -0.687 | 1.680 | 1.627 | 1.824 | 0.372 |
| A G | 0.816 | 0.8846 | 2.522 | 0.945 | -1.706 | 1.295 | 0.188 |
| B G | 1.600 | 0.991 | 0.648 | 1.053 | 0.952 | 1.446 | 0.51 |
| C G | 2.030 | 1.513 | 0.403 | 1.020 | 1.627 | 1.825 | 0.372 |
| D G | 0.809 | 0.511 | 3.244 | 53.148 | -2.435 | 53.152 | 0.963 |
| E F | -1.174 | 4.091 | 1.785 | 1.070 | -2.960 | 4.155 | 0.476 |
| E G | 2.808 | 0.979 | -0.088 | 5.484 | 2.896 | 5.471 | 0.597 |
| F G | 1.110 | 0.404 | 7.614 | 9.200 | -6.504 | 9.213 | 0.48 |

**BBS**

| **Side** | **Direct Coef.** | **Std. Err.** | **Indirect**  **Coef.** | **Std. Err.** | **Difference Coef.** | **Std. Err.** | ***P*>\|z\|** |
| --- | --- | --- | --- | --- | --- | --- | --- |
| A B | 3.2 | 2.767971 | 7.521317 | 4.260814 | -4.321317 | 5.080964 | 0.395 |
| A G | -2.0883 | 1.929109 | -6.03578 | 4.529968 | 3.947499 | 4.922736 | 0.423 |
| B G | -9.6000 | 3.804312 | -5.27805 | 3.368373 | -4.321942 | 5.081216 | 0.395 |
| C G | -2.1058 | 1.63083 | -5.41710 | 38.0036 | 3.311218 | 38.03764 | 0.931 |
| D G | -5.1815 | 1.862971 | -3.43157 | 180.0155 | -1.749962 | 180.0344 | 0.992 |
| E F | 2.55007 | 3.909854 | -0.01140 | 2.930543 | 2.561466 | 4.88622 | 0.600 |
| E G | -3.7999 | 2.266268 | -1.24579 | 4.324536 | -2.554083 | 4.882242 | 0.601 |
| F G | -3.7889 | 1.858443 | -6.34910 | 4.516742 | 2.5602 | 4.884108 | 0.600 |

**Depression scale**

| **Side** | **Direct**  **Coef.** | **Std. Err.** | **Indirect**  **Coef.** | **Std. Err.** | **Difference**  **Coef.** | **Std. Err.** | ***P*>\|z\|** |
| --- | --- | --- | --- | --- | --- | --- | --- |
| A F | .454196 | .268305 | .117700 | 11.9534 | .3364958 | 11.9564 | 0.978 |
| B F | .518531 | .572047 | .908080 | 63.2530 | -.3895492 | 63.2555 | 0.995 |
| C F | -.55845 | .309711 | .931456 | 44.7274 | -1.489911 | 44.7285 | 0.973 |
| D F | .412402 | .315979 | .856693 | 36.5206 | -.4442905 | 36.5223 | 0.990 |
| E F | .875653 | .387707 | .882667 | 63.2428 | -.0070139 | 63.2440 | 1.000 |

**Cognitive measurement**

| **Side** | **Direct**  **Coef.** | **Std. Err.** | **Indirect**  **Coef.** | **Std. Err.** | **Difference**  **Coef.** | **Std. Err.** | ***P*>z** |
| --- | --- | --- | --- | --- | --- | --- | --- |
| A F | -0.0162 | 0.37408 | -0.2329 | 10.5433 | 0.216602 | 10.550 | 0.984 |
| B F | 0.05722 | 0.70817 | -0.0483 | 63.2582 | 0.105541 | 63.263 | 0.999 |
| C F | 0.17918 | 0.46314 | -0.0291 | 44.7345 | 0.208364 | 44.737 | 0.996 |
| D F | -0.3677 | 0.35754 | 0.01906 | 31.6367 | -0.38683 | 31.639 | 0.991 |
| E F | -1.3223 | 0.61978 | 0.11289 | 44.7346 | -1.43522 | 44.739 | 0.974 |

**ADL**

| **Side** | **Direct**  **Coef.** | **Std. Err.** | **Indirect**  **Coef.** | **Std. Err.** | **Difference**  **Coef.** | **Std. Err.** | ***P*>z** |
| --- | --- | --- | --- | --- | --- | --- | --- |
| A C | 0.85129 | 0.24811 | -0.00939 | 0.24056 | 0.860678 | 0.34689 | 0.013 |
| A F | 0.41012 | 0.13290 | 1.48106 | 0.56383 | -1.07093 | 0.58017 | 0.065 |
| B F | 0.52978 | 0.22567 | 0.93644 | 63.2592 | -0.40665 | 63.2596 | 0.995 |
| C E | -0.34414 | 0.40757 | -0.36836 | 0.36889 | 0.024215 | 0.54972 | 0.965 |
| C F | 0.16423 | 0.16306 | -0.4444 | 0.38426 | 0.608624 | 0.41171 | 0.139 |
| D F | 0.59885 | 0.20408 | 0.88736 | 44.7227 | -0.28851 | 44.7232 | 0.995 |
| E F | 0.448979 | 0.325807 | 0.424868 | 0.443356 | 0.024111 | 0.5497 | 0.965 |

# Supplementary Table 3. Mean rank and SUCRA

Supplementary Table 3a: mean rank of varied methods of therapies for PD in different outcomes.

|  | **Depression** | **Cognition** | **ADL** |
| --- | --- | --- | --- |
| Treadmill | 2.9 | 4.0 | 2.8 |
| Walking | 2.8 | 4.1 | / |
| Cycling | 5.9 | 4.6 | 2.5 |
| Dance | 3.0 | 2.8 | 5.1 |
| Yoga | 1.7 | 1.2 | 2.0 |
| Taichi | / | / | 3.0 |
| Control | 4.7 | 4.2 | 5.6 |

Supplementary Table 3b: SUCRA (Surface under the cumulative ranking curve) of varied methods of therapies for PD in different outcomes.

|  | **Depression** | **Cognition** | **ADL** |
| --- | --- | --- | --- |
| Treadmill | 62.7 | 39.3 | 64.7 |
| Walking | 63.3 | 37.3 | / |
| Cycling | 2.1 | 27.7 | 70.7 |
| Dance | 59.5 | 64.4 | 17.2 |
| Yoga | 86.3 | 95.1 | 79.5 |
| Taichi | / | / | 60.4 |
| Control | 26.1 | 36.3 | 7.5 |

# Supplementary Figure 1. The flowchart of screening process


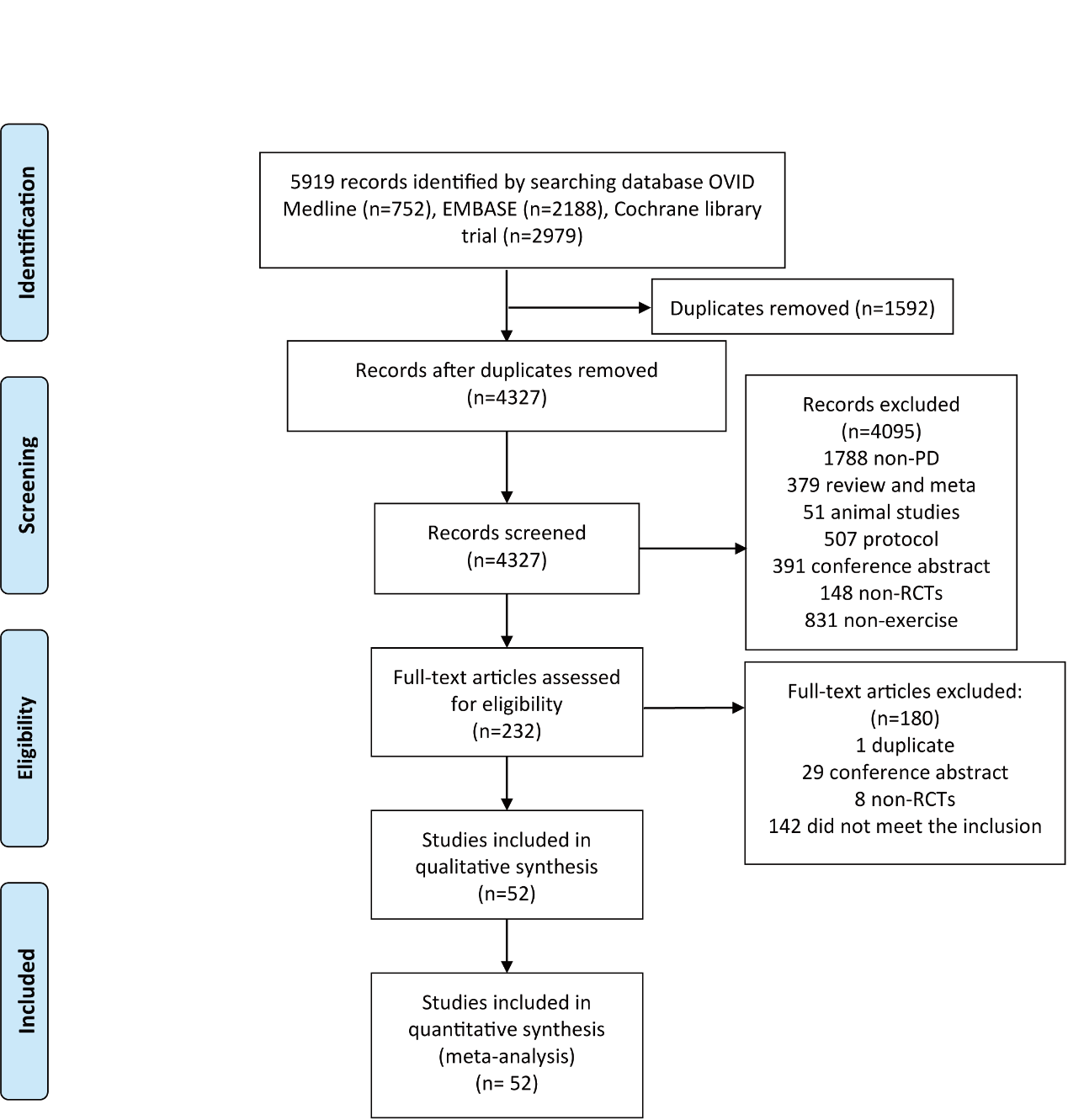


Figure 1. The flowchart of screening process

# Supplementary Figure 2. Risk of bias summary


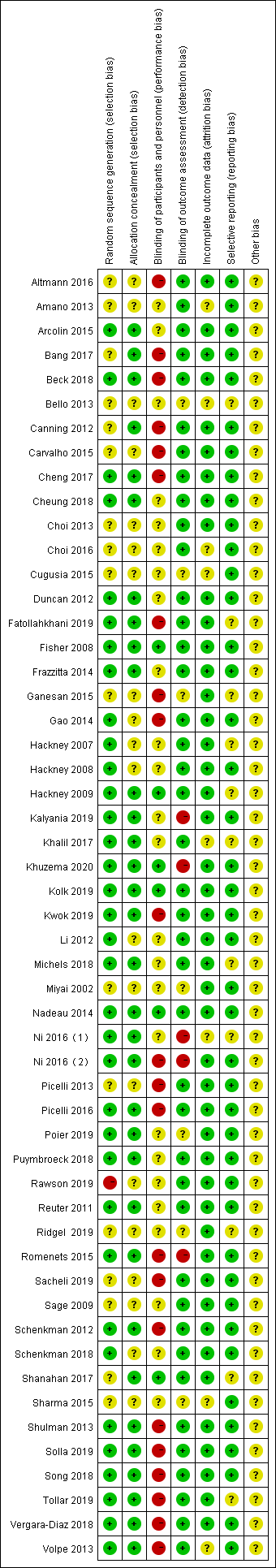


Supplementary Figure 2. Risk of bias summary

# Supplementary Figure 3. Risk of bias graph


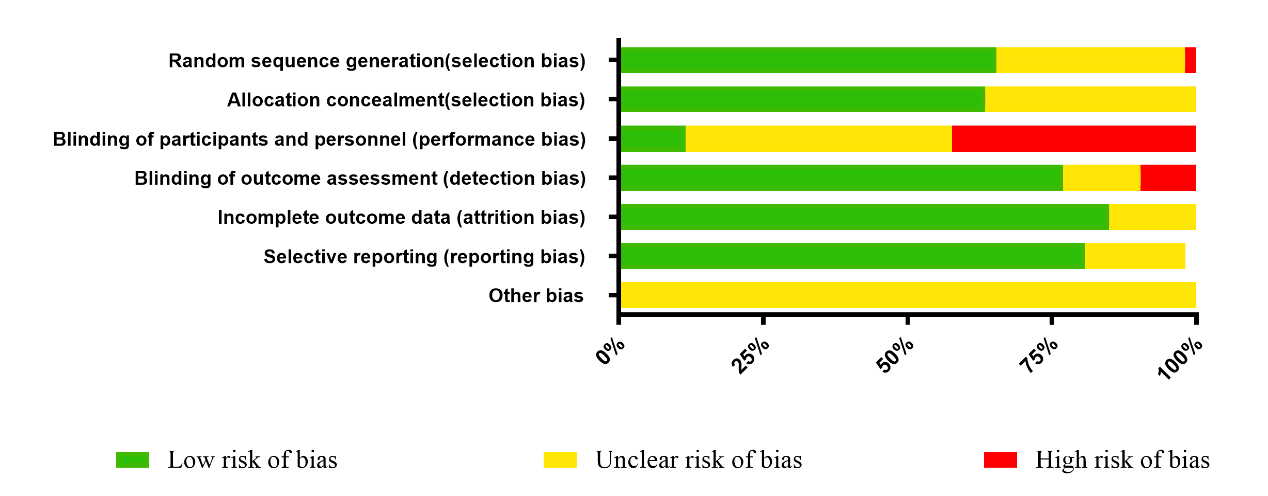
Supplementary Figure 3. Risk of bias graph

# Supplementary Figure 4. Network plot


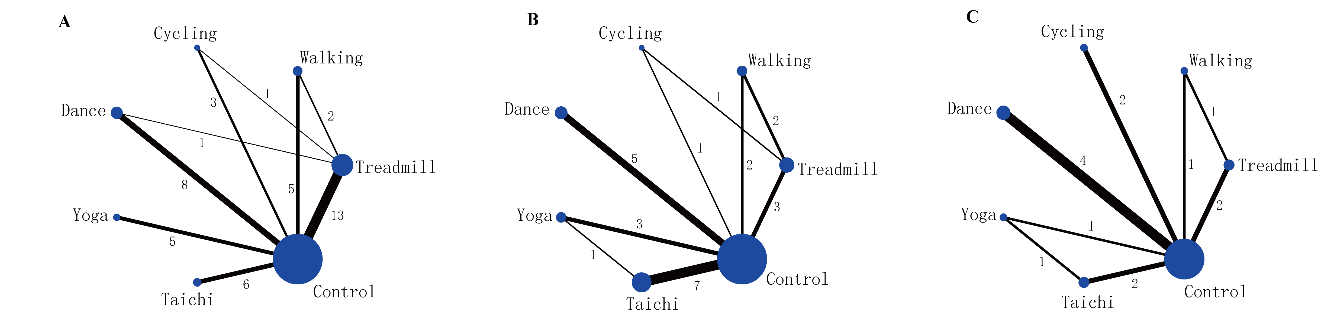


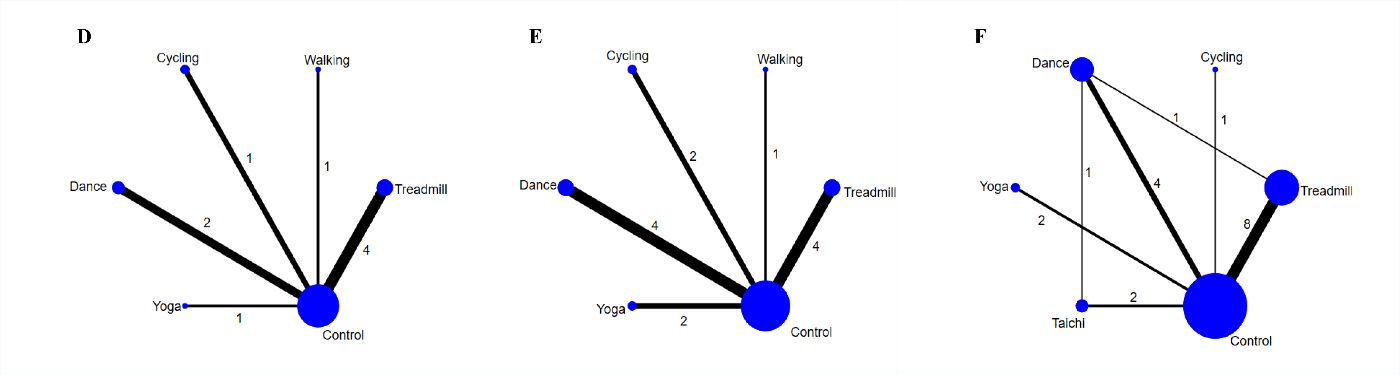


Supplementary Figure 4. The network plot of the studies exploring the efficacy of therapies for PD in different outcome. Each node represents one kind of therapy and the node size indicates the number of trails in this therapy were studied. The line between two nodes represents head to head comparison. The thickness of lines related to the number of trails that assess the comparisons. Figure 4-A, the network plot in UPDRS-motor outcome; Figure 4-B the network plot in TUG outcome; Figure 4-C, the network plot in BBS outcome. Figure 4-D, the network plot in depression outcome; Figure 4-E the network plot in cognitive outcome; Figure 4-F, the network plot in ADL outcome.

# Supplementary Figure 5. Results of meta-analysis of direct comparison

Estimated are presented as mean difference (or SMD) and 95% confidence intervals.

**Supplementary Figure 5A-C: Pairwise meta-analysis of standardized mean difference (95% CI) in non-motor outcomes**

**D**

**E**


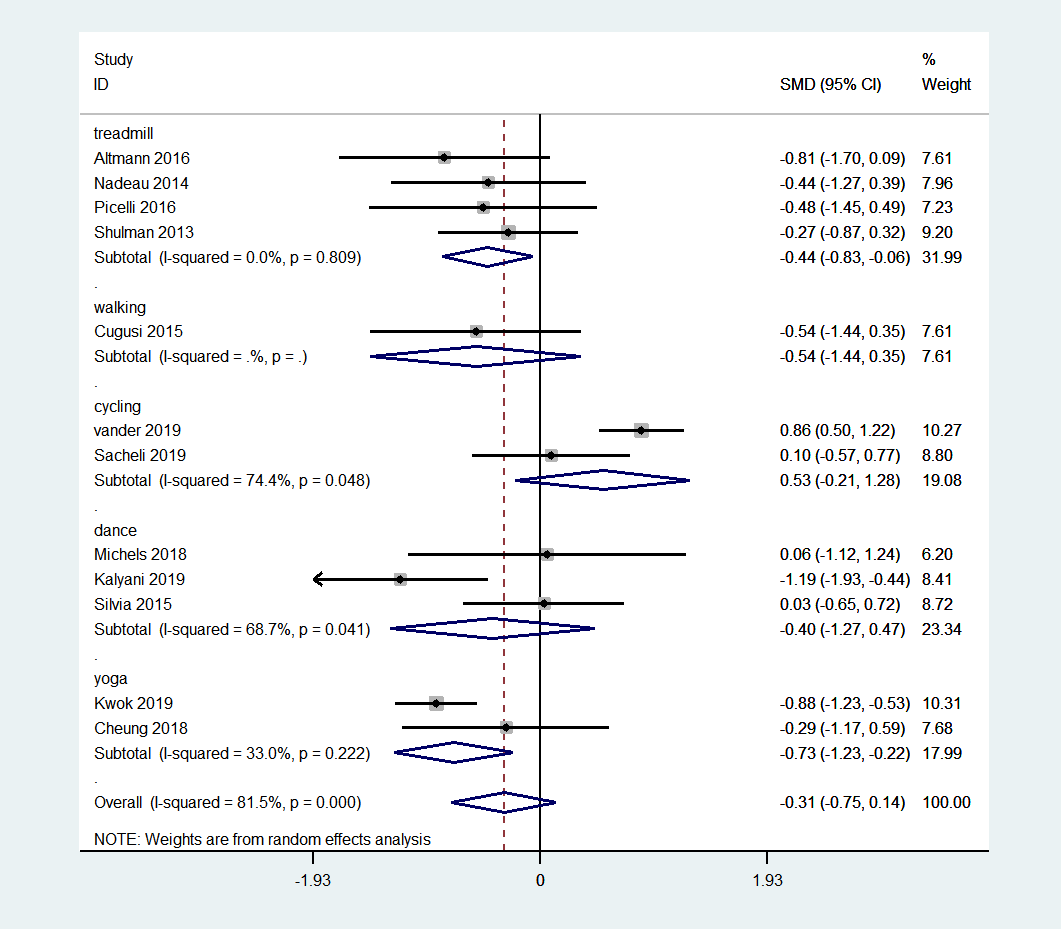

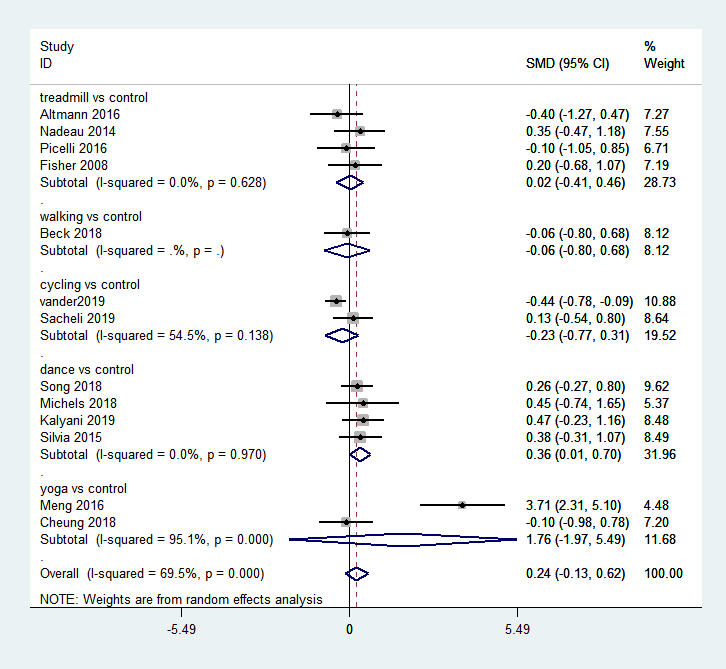


**Depression scale (treatment vs control)**

**Cognitive scale (treatment vs control)**


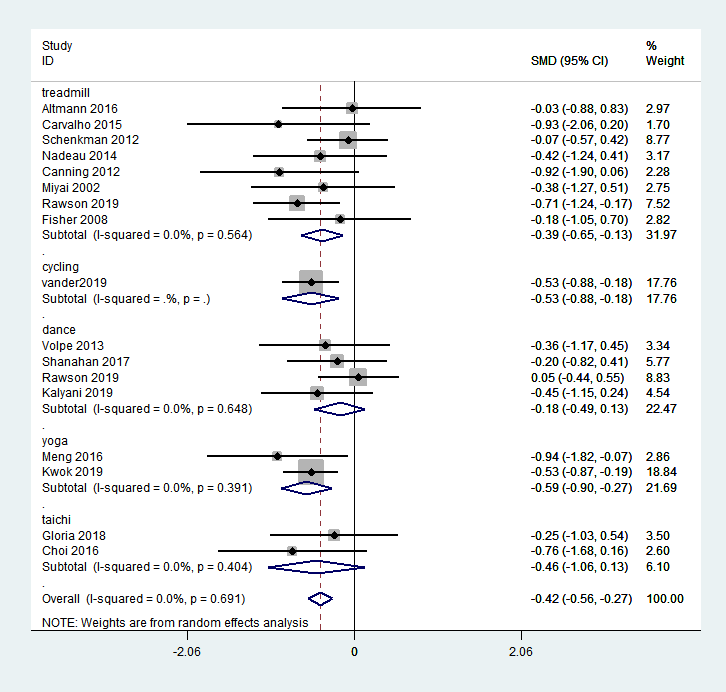


**F**

**ADL (treatment vs control)**

# Supplementary Figure 6. Results of network meta-analysis

Supplementary Figure 6-A: Network meta-analysis of the efficacy of therapies in depression outcome


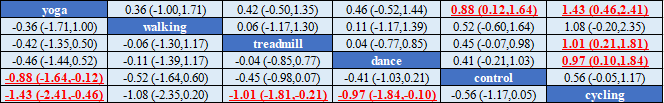


Supplementary Figure 6-B: Network meta-analysis of the efficacy of therapies in cognitive outcome


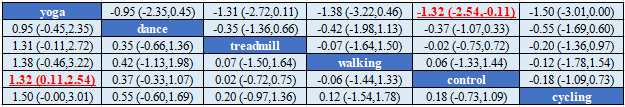


Supplementary Figure 6-C: Network meta-analysis of the efficacy of therapies in ADL outcome


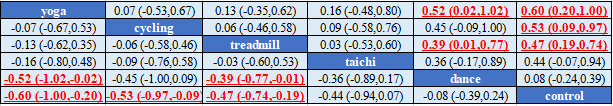


Supplementary Figure 6. Network meta-analysis of the efficacy of therapies in different outcome. Mean difference (MD) and 95% credible interval (CrI) estimation were calculated as column-defining intervention compared the row-defining intervention. Significant results were labeled with bold, red and underlined.

# Supplementary Figure 7. Rank plot: cumulative rank of varied treatments for PD in non-motor outcomes.

**
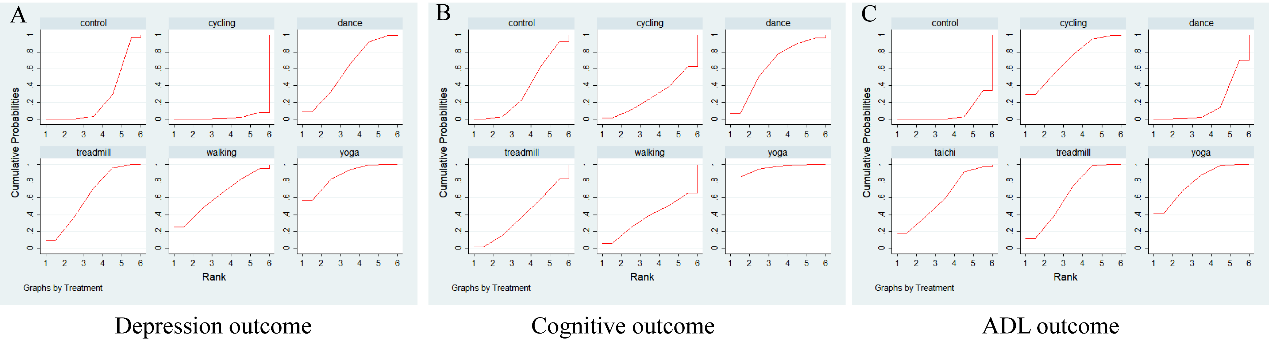
**

Supplementary Figure 7. Rank plot: cumulative rank of varied treatments for PD in non-motor outcomes.

# Supplementary Figure 8. Sensitivity analysis of motor outcomes

Supplementary Figure 8A-D: Results of sensitivity analysis by only including trials with early-to-moderate stage of Parkinson disease

**
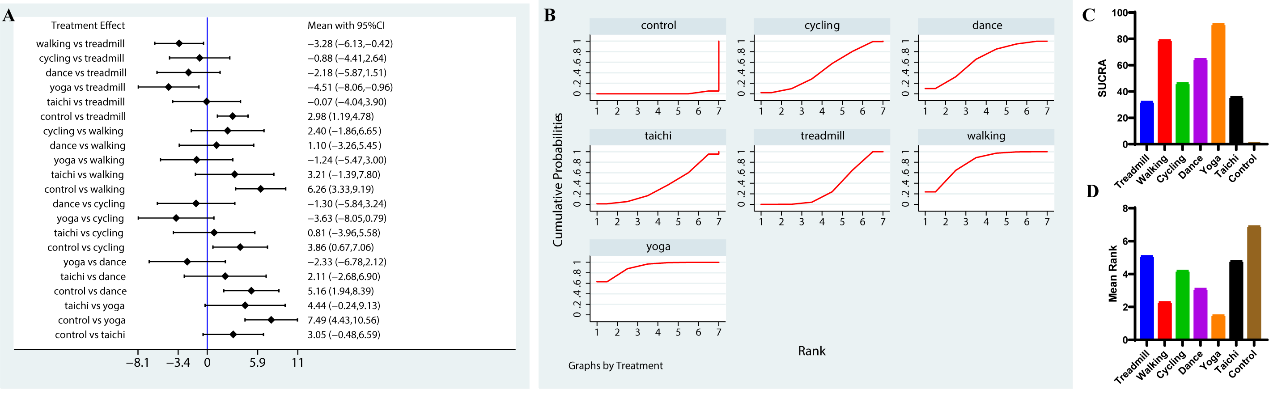
**

Note: figure8A: MD (Mean difference) with 95%CI of network meta-analysis for UPDRS-motor scale; figure8B-D: Rank plot: cumulative rank of varied treatments for PD in UPDRS-motor outcomes.

Supplementary Figure 8E-H: Results of sensitivity analysis by excluding studies that contained resistance active exercise as control group

**
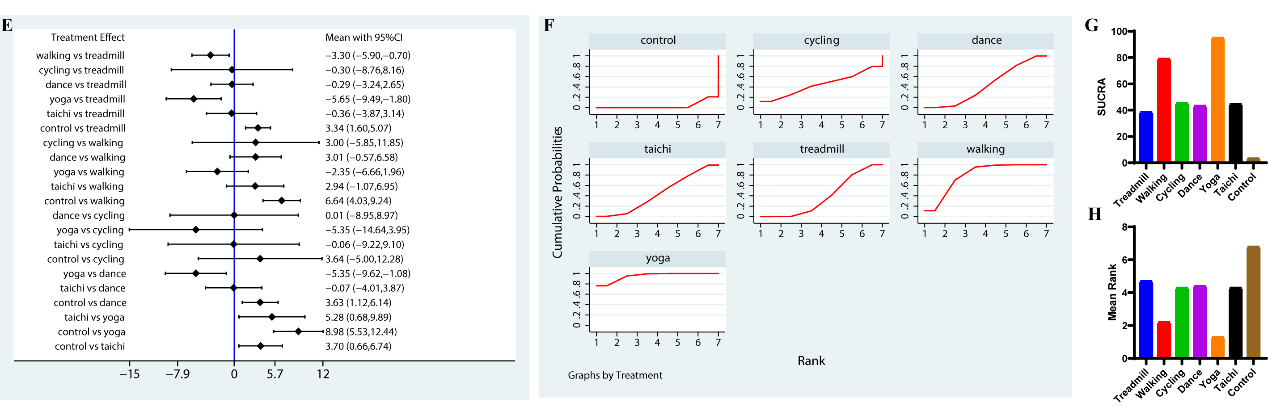
**

Note: figure8E: MD (Mean difference) with 95%CI of network meta-analysis for UPDRS-motor scale; figure8F-H: Rank plot: cumulative rank of varied treatments for PD in UPDRS-motor outcomes.

Supplementary Figure 8I-L: Results of sensitivity analysis by only including trials with early-to-moderate stage of Parkinson disease

**
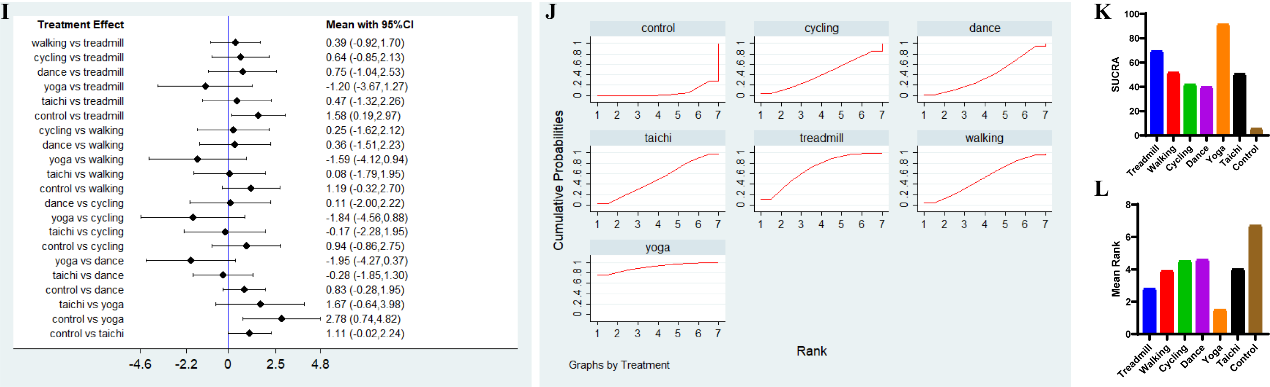
**

Note: figure8I: MD (Mean difference) with 95%CI of network meta-analysis for TUG scale; figure8J-L: Rank plot: cumulative rank of varied treatments for PD in TUG outcomes.

Supplementary Figure 8M-P: Results of sensitivity analysis by excluding studies that contained resistance active exercise as control group
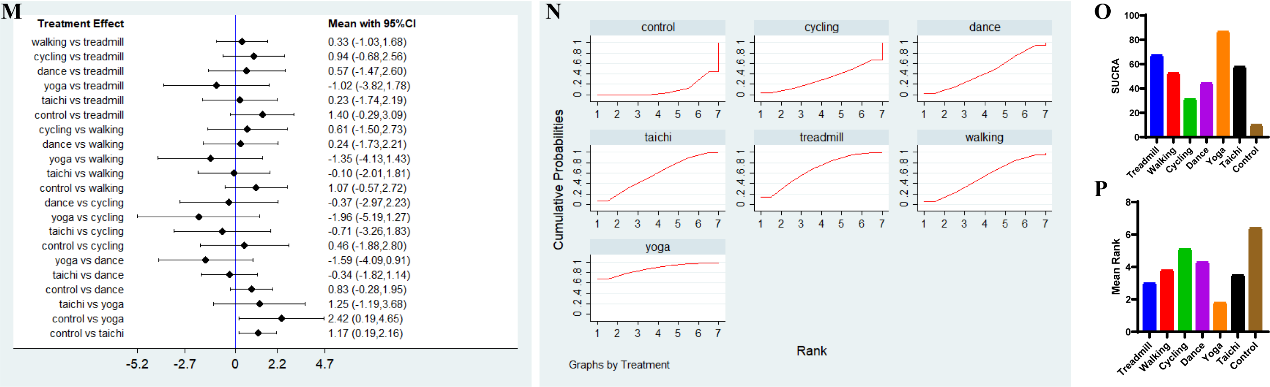


Note: figure8M: MD (Mean difference) with 95%CI of network meta-analysis for TUG scale; figure8N-P: Rank plot: cumulative rank of varied treatments for PD in TUG outcomes.

Supplementary Figure 8Q-T: Results of sensitivity analysis by only including trials with early-to-moderate stage of Parkinson disease


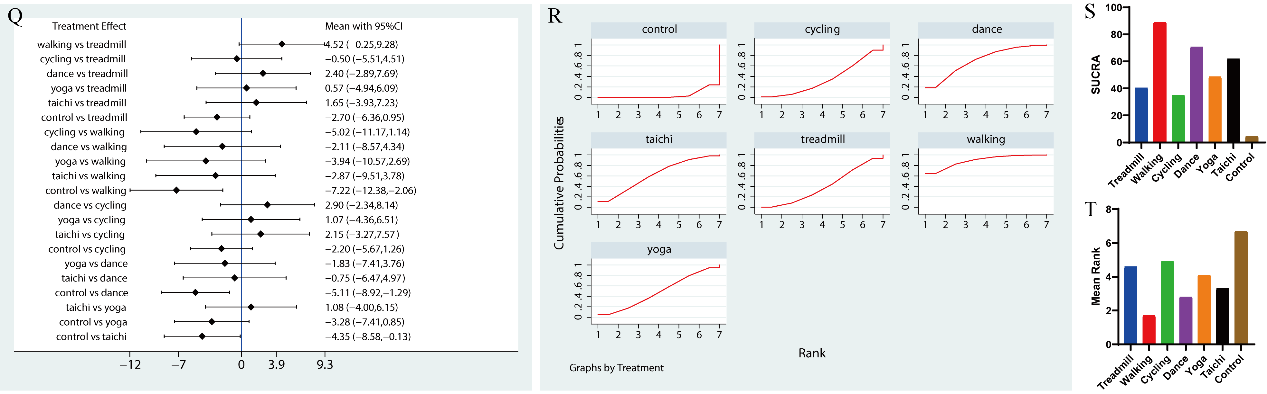


Note: figure8Q: MD (Mean difference) with 95%CI of network meta-analysis for BBS scale; figure8R-T: Rank plot: cumulative rank of varied treatments for PD in BBS outcomes.

Supplementary Figure 8U-X: Results of sensitivity analysis by excluding studies that contained resistance active exercise as control group


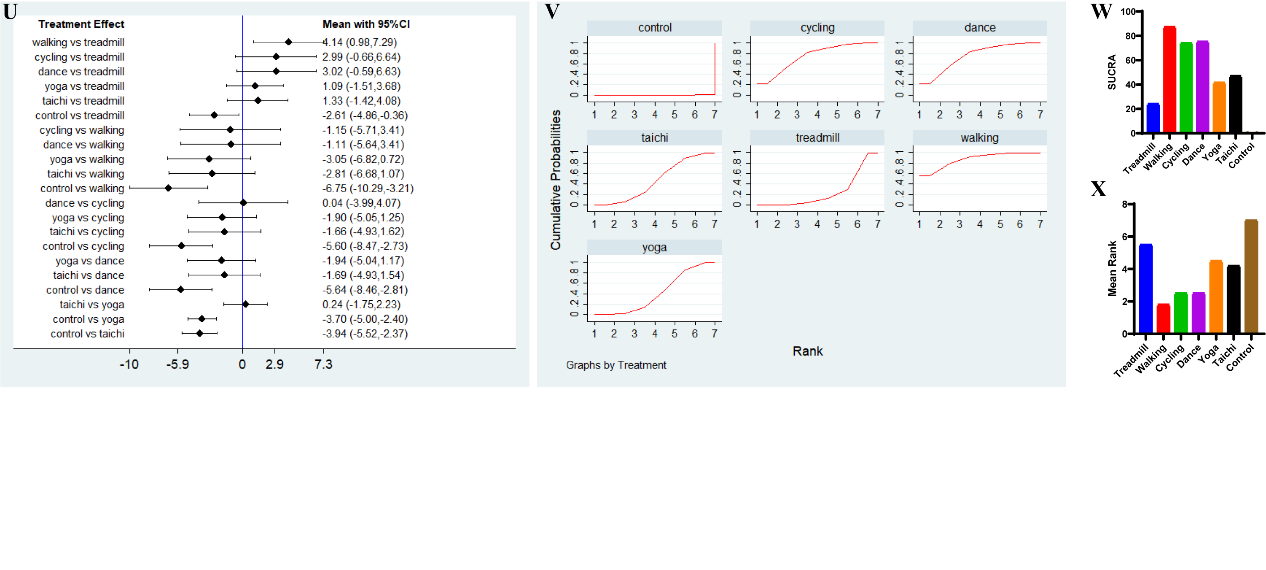


Note: figure8U: MD (Mean difference) with 95%CI of network meta-analysis for BBS scale; figure8V-X: Rank plot: cumulative rank of varied treatments for PD in BBS outcomes.

# Supplementary Figure 9. Sensitivity analysis of non-motor outcomes

Supplementary Figure 9A-D: Results of sensitivity analysis by only including trials with early-to-moderate stage of Parkinson disease

**
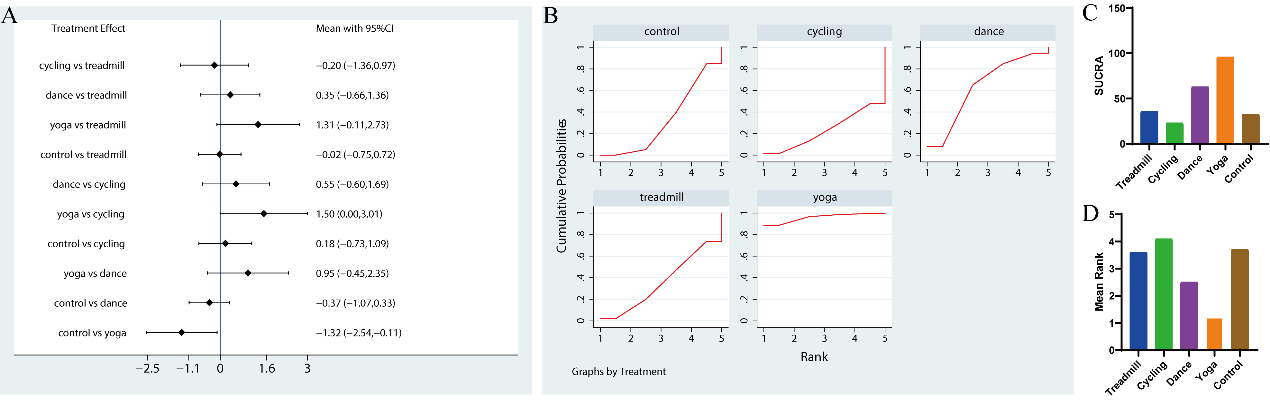
**

Note: figure9A: MD (Mean difference) with 95%CI of network meta-analysis for cognitive scale; figure9B-D: Rank plot: cumulative rank of varied treatments for PD in cognitive outcomes.

Supplementary Figure 9E-H: Results of sensitivity analysis by excluding studies that contained resistance active exercise as control group

**
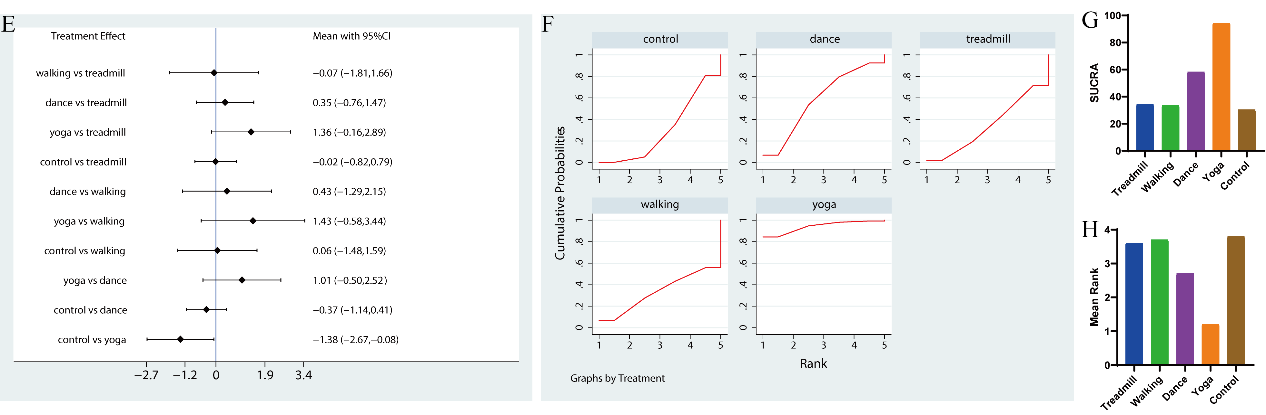
**

Note: figure9E: MD (Mean difference) with 95%CI of network meta-analysis for cognitive scale; figure9F-H: Rank plot: cumulative rank of varied treatments for PD in cognitive outcomes.

Supplementary Figure 9I-L: Results of sensitivity analysis by only including trials with early-to-moderate stage of Parkinson disease

**
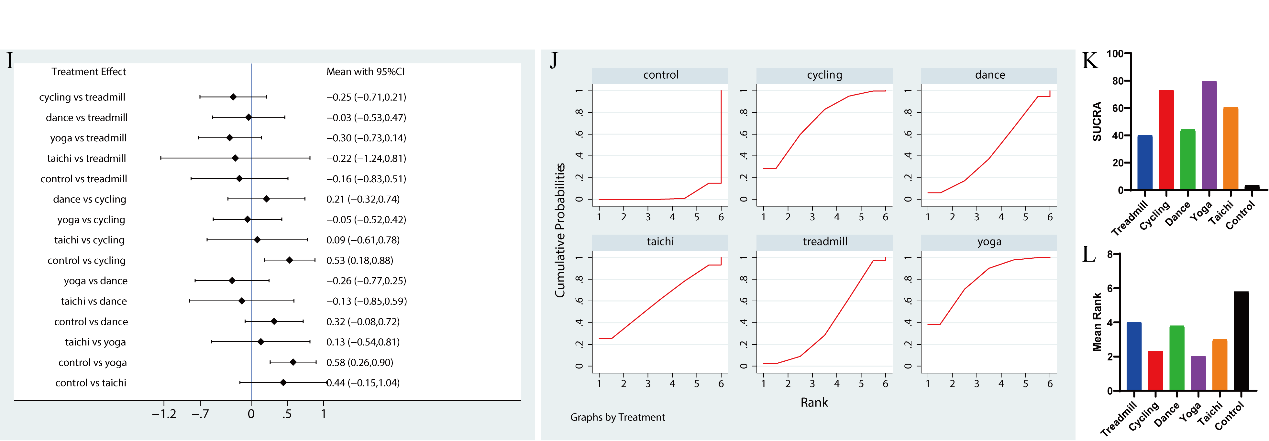
**

Note: figure9I: MD (Mean difference) with 95%CI of network meta-analysis for ADL-scale; figure9J-L: Rank plot: cumulative rank of varied treatments for PD in ADL outcomes.

Supplementary Figure 9E-H: Results of sensitivity analysis by excluding studies that contained resistance active exercise as control group

**
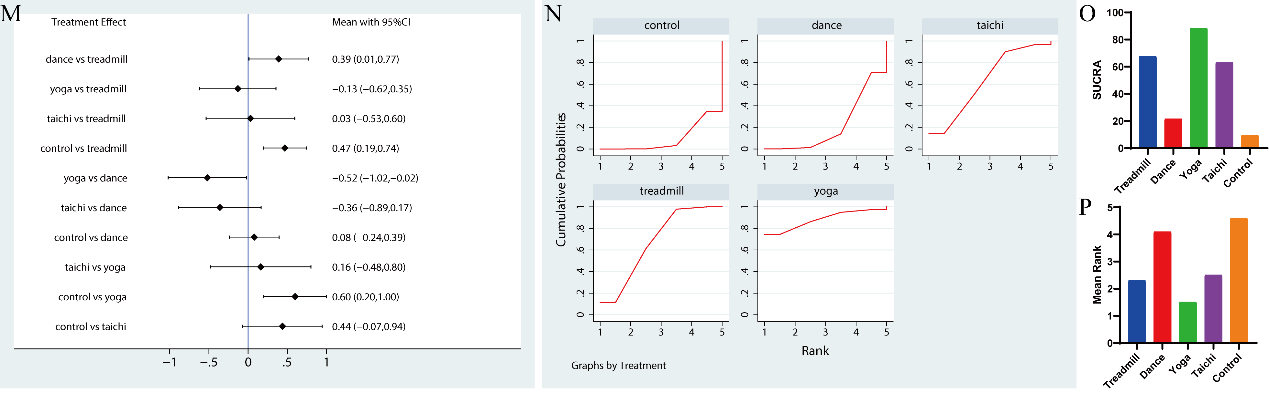
**

Note: figure9M: MD (Mean difference) with 95%CI of network meta-analysis for ADL scale; figure9N-P: Rank plot: cumulative rank of varied treatments for PD in ADL outcomes.

# Supplementary Figure 10 Comparison-adjusted funnel plot for the network of the outcomes.

Figure 10A-C Comparison-adjusted funnel plot for the network of motor outcomes:


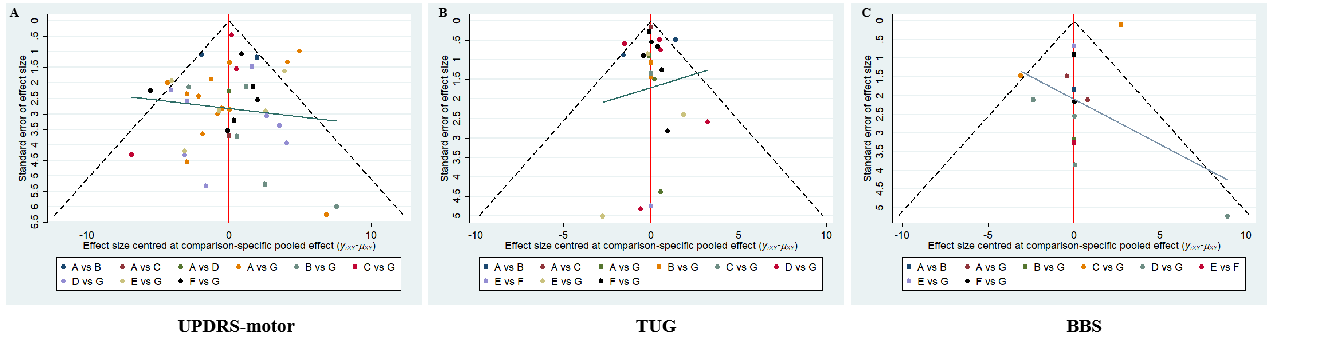


Supplementary Figure 10A-C The funnel plot for the network of the motor outcomes. A-G represents different intervention: A: treadmill; B: walking; C: cycling; D: dance; E: yoga; F: tai chi; G: control

Figure 10D-F Comparison-adjusted funnel plot for the network of non-motor outcomes:


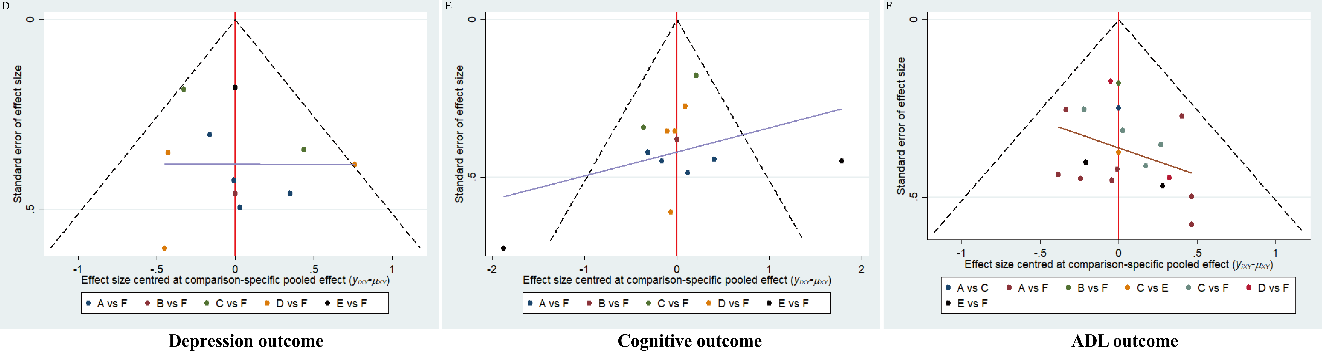


Supplementary Figure 10D-F The funnel plot for the network of the non-motor outcomes. A-F represents different intervention: Figure A: A: treadmill; B: walking; C: cycling; D: dance; E: yoga; F: control; Figure B: A: treadmill; B: walking; C: cycling; D: dance; E: yoga; F: control; Figure C: A: treadmill; B: cycling; C: dance; D: yoga; E: tai chi; F: control

# Supplementary references

Altmann, L.J., Stegemöller, E., Hazamy, A.A., Wilson, J.P., Bowers, D., Okun, M.S., et al. (2016). Aerobic Exercise Improves Mood, Cognition, and Language Function in Parkinson's Disease: Results of a Controlled Study. *J Int Neuropsychol Soc* 22(9)**,** 878-889. doi: 10.1017/s135561771600076x.

Amano, S., Nocera, J.R., Vallabhajosula, S., Juncos, J.L., Gregor, R.J., Waddell, D.E., et al. (2013). The effect of Tai Chi exercise on gait initiation and gait performance in persons with Parkinson's disease. *Parkinsonism Relat Disord* 19(11)**,** 955-960. doi: 10.1016/j.parkreldis.2013.06.007.

Arcolin, I., Pisano, F., Delconte, C., Godi, M., Schieppati, M., Mezzani, A., et al. (2016). Intensive cycle ergometer training improves gait speed and endurance in patients with Parkinson's disease: A comparison with treadmill training. *Restor Neurol Neurosci* 34(1)**,** 125-138. doi: 10.3233/rnn-150506.

Arfa-Fatollahkhani, P., Safar Cherati, A., Habibi, S.A.H., Shahidi, G.A., Sohrabi, A., and Zamani, B. (2019). Effects of treadmill training on the balance, functional capacity and quality of life in Parkinson's disease: A randomized clinical trial. *J Complement Integr Med* 17(1). doi: 10.1515/jcim-2018-0245.

Bang, D.H., and Shin, W.S. (2017). Effects of an intensive Nordic walking intervention on the balance function and walking ability of individuals with Parkinson's disease: a randomized controlled pilot trial. *Aging Clin Exp Res* 29(5)**,** 993-999. doi: 10.1007/s40520-016-0648-9.

Beck, E.N., Intzandt, B.N., and Almeida, Q.J. (2018). Can Dual Task Walking Improve in Parkinson's Disease After External Focus of Attention Exercise? A Single Blind Randomized Controlled Trial. *Neurorehabil Neural Repair* 32(1)**,** 18-33. doi: 10.1177/1545968317746782.

Bello, O., Sanchez, J.A., Lopez-Alonso, V., Márquez, G., Morenilla, L., Castro, X., et al. (2013). The effects of treadmill or overground walking training program on gait in Parkinson's disease. *Gait Posture* 38(4)**,** 590-595. doi: 10.1016/j.gaitpost.2013.02.005.

Canning, C.G., Allen, N.E., Dean, C.M., Goh, L., and Fung, V.S. (2012). Home-based treadmill training for individuals with Parkinson's disease: a randomized controlled pilot trial. *Clin Rehabil* 26(9)**,** 817-826. doi: 10.1177/0269215511432652.

Carvalho, A., Barbirato, D., Araujo, N., Martins, J.V., Cavalcanti, J.L., Santos, T.M., et al. (2015). Comparison of strength training, aerobic training, and additional physical therapy as supplementary treatments for Parkinson's disease: pilot study. *Clin Interv Aging* 10**,** 183-191. doi: 10.2147/cia.S68779.

Cheng, F.Y., Yang, Y.R., Wu, Y.R., Cheng, S.J., and Wang, R.Y. (2017). Effects of curved-walking training on curved-walking performance and freezing of gait in individuals with Parkinson's disease: A randomized controlled trial. *Parkinsonism Relat Disord* 43**,** 20-26. doi: 10.1016/j.parkreldis.2017.06.021.

Cheung, C., Bhimani, R., Wyman, J.F., Konczak, J., Zhang, L., Mishra, U., et al. (2018). Effects of yoga on oxidative stress, motor function, and non-motor symptoms in Parkinson's disease: a pilot randomized controlled trial. *Pilot Feasibility Stud* 4**,** 162. doi: 10.1186/s40814-018-0355-8.

Choi, H.J. (2016). Effects of therapeutic Tai chi on functional fitness and activities of daily living in patients with Parkinson disease. *J Exerc Rehabil* 12(5)**,** 499-503. doi: 10.12965/jer.1632654.327.

Choi, H.J., Garber, C.E., Jun, T.W., Jin, Y.S., Chung, S.J., and Kang, H.J. (2013). Therapeutic effects of tai chi in patients with Parkinson's disease. *ISRN Neurol* 2013**,** 548240. doi: 10.1155/2013/548240.

Cugusi, L., Solla, P., Serpe, R., Carzedda, T., Piras, L., Oggianu, M., et al. (2015). Effects of a Nordic Walking program on motor and non-motor symptoms, functional performance and body composition in patients with Parkinson's disease. *NeuroRehabilitation* 37(2)**,** 245-254. doi: 10.3233/nre-151257.

Duncan, R.P., and Earhart, G.M. (2012). Randomized controlled trial of community-based dancing to modify disease progression in Parkinson disease. *Neurorehabil Neural Repair* 26(2)**,** 132-143. doi: 10.1177/1545968311421614.

Fisher, B.E., Wu, A.D., Salem, G.J., Song, J., Lin, C.H., Yip, J., et al. (2008). The effect of exercise training in improving motor performance and corticomotor excitability in people with early Parkinson's disease. *Arch Phys Med Rehabil* 89(7)**,** 1221-1229. doi: 10.1016/j.apmr.2008.01.013.

Frazzitta, G., Maestri, R., Ghilardi, M.F., Riboldazzi, G., Perini, M., Bertotti, G., et al. (2014). Intensive rehabilitation increases BDNF serum levels in parkinsonian patients: a randomized study. *Neurorehabil Neural Repair* 28(2)**,** 163-168. doi: 10.1177/1545968313508474.

Ganesan, M., Sathyaprabha, T.N., Pal, P.K., and Gupta, A. (2015). Partial Body Weight-Supported Treadmill Training in Patients With Parkinson Disease: Impact on Gait and Clinical Manifestation. *Arch Phys Med Rehabil* 96(9)**,** 1557-1565. doi: 10.1016/j.apmr.2015.05.007.

Gao, Q., Leung, A., Yang, Y., Wei, Q., Guan, M., Jia, C., et al. (2014). Effects of Tai Chi on balance and fall prevention in Parkinson's disease: a randomized controlled trial. *Clin Rehabil* 28(8)**,** 748-753. doi: 10.1177/0269215514521044.

Hackney, M.E., and Earhart, G.M. (2008). Tai Chi improves balance and mobility in people with Parkinson disease. *Gait Posture* 28(3)**,** 456-460. doi: 10.1016/j.gaitpost.2008.02.005.

Hackney, M.E., and Earhart, G.M. (2009). Effects of dance on movement control in Parkinson's disease: a comparison of Argentine tango and American ballroom. *J Rehabil Med* 41(6)**,** 475-481. doi: 10.2340/16501977-0362.

Kalyani, H.H.N., Sullivan, K.A., Moyle, G., Brauer, S., Jeffrey, E.R., and Kerr, G.K. (2019). Impacts of dance on cognition, psychological symptoms and quality of life in Parkinson's disease. *NeuroRehabilitation* 45(2)**,** 273-283. doi: 10.3233/nre-192788.

Khalil, H., Busse, M., Quinn, L., Nazzal, M., Batyha, W., Alkhazaleh, S., et al. (2017). A pilot study of a minimally supervised home exercise and walking program for people with Parkinson's disease in Jordan. *Neurodegener Dis Manag* 7(1)**,** 73-84. doi: 10.2217/nmt-2016-0041.

Khuzema, A., Brammatha, A., and Arul Selvan, V. (2020). Effect of home-based Tai Chi, Yoga or conventional balance exercise on functional balance and mobility among persons with idiopathic Parkinson's disease: An experimental study. *Hong Kong Physiother J* 40(1)**,** 39-49. doi: 10.1142/s1013702520500055.

Kwok, J.Y.Y., Kwan, J.C.Y., Auyeung, M., Mok, V.C.T., Lau, C.K.Y., Choi, K.C., et al. (2019). Effects of Mindfulness Yoga vs Stretching and Resistance Training Exercises on Anxiety and Depression for People With Parkinson Disease: A Randomized Clinical Trial. *JAMA Neurol* 76(7)**,** 755-763. doi: 10.1001/jamaneurol.2019.0534.

Li, F., Harmer, P., Fitzgerald, K., Eckstrom, E., Stock, R., Galver, J., et al. (2012). Tai chi and postural stability in patients with Parkinson's disease. *N Engl J Med* 366(6)**,** 511-519. doi: 10.1056/NEJMoa1107911.

Michels, K., Dubaz, O., Hornthal, E., and Bega, D. (2018). "Dance Therapy" as a psychotherapeutic movement intervention in Parkinson's disease. *Complement Ther Med* 40**,** 248-252. doi: 10.1016/j.ctim.2018.07.005.

Miyai, I., Fujimoto, Y., Yamamoto, H., Ueda, Y., Saito, T., Nozaki, S., et al. (2002). Long-term effect of body weight-supported treadmill training in Parkinson's disease: a randomized controlled trial. *Arch Phys Med Rehabil* 83(10)**,** 1370-1373. doi: 10.1053/apmr.2002.34603.

Nadeau, A., Pourcher, E., and Corbeil, P. (2014). Effects of 24 wk of treadmill training on gait performance in Parkinson's disease. *Med Sci Sports Exerc* 46(4)**,** 645-655. doi: 10.1249/mss.0000000000000144.

Ni, M., Mooney, K., and Signorile, J.F. (2016a). Controlled pilot study of the effects of power yoga in Parkinson's disease. *Complement Ther Med* 25**,** 126-131. doi: 10.1016/j.ctim.2016.01.007.

Ni, M., Signorile, J.F., Mooney, K., Balachandran, A., Potiaumpai, M., Luca, C., et al. (2016b). Comparative Effect of Power Training and High-Speed Yoga on Motor Function in Older Patients With Parkinson Disease. *Arch Phys Med Rehabil* 97(3)**,** 345-354.e315. doi: 10.1016/j.apmr.2015.10.095.

Picelli, A., Melotti, C., Origano, F., Neri, R., Waldner, A., and Smania, N. (2013). Robot-assisted gait training versus equal intensity treadmill training in patients with mild to moderate Parkinson's disease: a randomized controlled trial. *Parkinsonism Relat Disord* 19(6)**,** 605-610. doi: 10.1016/j.parkreldis.2013.02.010.

Picelli, A., Varalta, V., Melotti, C., Zatezalo, V., Fonte, C., Amato, S., et al. (2016). Effects of treadmill training on cognitive and motor features of patients with mild to moderate Parkinson's disease: a pilot, single-blind, randomized controlled trial. *Funct Neurol* 31(1)**,** 25-31. doi: 10.11138/fneur/2016.31.1.025.

Poier, D., Rodrigues Recchia, D., Ostermann, T., and Büssing, A. (2019). A Randomized Controlled Trial to Investigate the Impact of Tango Argentino versus Tai Chi on Quality of Life in Patients with Parkinson Disease: A Short Report. *Complement Med Res* 26(6)**,** 398-403. doi: 10.1159/000500070.

Rawson, K.S., McNeely, M.E., Duncan, R.P., Pickett, K.A., Perlmutter, J.S., and Earhart, G.M. (2019). Exercise and Parkinson Disease: Comparing Tango, Treadmill, and Stretching. *J Neurol Phys Ther* 43(1)**,** 26-32. doi: 10.1097/npt.0000000000000245.

Reuter, I., Mehnert, S., Leone, P., Kaps, M., Oechsner, M., and Engelhardt, M. (2011). Effects of a flexibility and relaxation programme, walking, and nordic walking on Parkinson's disease. *J Aging Res* 2011**,** 232473. doi: 10.4061/2011/232473.

Ridgel, A.L., and Ault, D.L. (2019). High-Cadence Cycling Promotes Sustained Improvement in Bradykinesia, Rigidity, and Mobility in Individuals with Mild-Moderate Parkinson's Disease. *Parkinsons Dis* 2019**,** 4076862. doi: 10.1155/2019/4076862.

Rios Romenets, S., Anang, J., Fereshtehnejad, S.M., Pelletier, A., and Postuma, R. (2015). Tango for treatment of motor and non-motor manifestations in Parkinson's disease: a randomized control study. *Complement Ther Med* 23(2)**,** 175-184. doi: 10.1016/j.ctim.2015.01.015.

Sacheli, M.A., Neva, J.L., Lakhani, B., Murray, D.K., Vafai, N., Shahinfard, E., et al. (2019). Exercise increases caudate dopamine release and ventral striatal activation in Parkinson's disease. *Mov Disord* 34(12)**,** 1891-1900. doi: 10.1002/mds.27865.

Sage, M.D., and Almeida, Q.J. (2009). Symptom and gait changes after sensory attention focused exercise vs aerobic training in Parkinson's disease. *Mov Disord* 24(8)**,** 1132-1138. doi: 10.1002/mds.22469.

Schenkman, M., Hall, D.A., Barón, A.E., Schwartz, R.S., Mettler, P., and Kohrt, W.M. (2012). Exercise for people in early- or mid-stage Parkinson disease: a 16-month randomized controlled trial. *Phys Ther* 92(11)**,** 1395-1410. doi: 10.2522/ptj.20110472.

Schenkman, M., Moore, C.G., Kohrt, W.M., Hall, D.A., Delitto, A., Comella, C.L., et al. (2018). Effect of High-Intensity Treadmill Exercise on Motor Symptoms in Patients With De Novo Parkinson Disease: A Phase 2 Randomized Clinical Trial. *JAMA Neurol* 75(2)**,** 219-226. doi: 10.1001/jamaneurol.2017.3517.

Shanahan, J., Morris, M.E., Bhriain, O.N., Volpe, D., Lynch, T., and Clifford, A.M. (2017). Dancing for Parkinson Disease: A Randomized Trial of Irish Set Dancing Compared With Usual Care. *Arch Phys Med Rehabil* 98(9)**,** 1744-1751. doi: 10.1016/j.apmr.2017.02.017.

Sharma, N.K., Robbins, K., Wagner, K., and Colgrove, Y.M. (2015). A randomized controlled pilot study of the therapeutic effects of yoga in people with Parkinson's disease. *Int J Yoga* 8(1)**,** 74-79. doi: 10.4103/0973-6131.146070.

Shulman, L.M., Katzel, L.I., Ivey, F.M., Sorkin, J.D., Favors, K., Anderson, K.E., et al. (2013). Randomized clinical trial of 3 types of physical exercise for patients with Parkinson disease. *JAMA Neurol* 70(2)**,** 183-190. doi: 10.1001/jamaneurol.2013.646.

Solla, P., Cugusi, L., Bertoli, M., Cereatti, A., Della Croce, U., Pani, D., et al. (2019). Sardinian Folk Dance for Individuals with Parkinson's Disease: A Randomized Controlled Pilot Trial. *J Altern Complement Med* 25(3)**,** 305-316. doi: 10.1089/acm.2018.0413.

Song, J., Paul, S.S., Caetano, M.J.D., Smith, S., Dibble, L.E., Love, R., et al. (2018). Home-based step training using videogame technology in people with Parkinson's disease: a single-blinded randomised controlled trial. *Clin Rehabil* 32(3)**,** 299-311. doi: 10.1177/0269215517721593.

Tollár, J., Nagy, F., and Hortobágyi, T. (2019). Vastly Different Exercise Programs Similarly Improve Parkinsonian Symptoms: A Randomized Clinical Trial. *Gerontology* 65(2)**,** 120-127. doi: 10.1159/000493127.

van der Kolk, N.M., de Vries, N.M., Kessels, R.P.C., Joosten, H., Zwinderman, A.H., Post, B., et al. (2019). Effectiveness of home-based and remotely supervised aerobic exercise in Parkinson's disease: a double-blind, randomised controlled trial. *Lancet Neurol* 18(11)**,** 998-1008. doi: 10.1016/S1474-4422(19)30285-6.

Van Puymbroeck, M., Walter, A.A., Hawkins, B.L., Sharp, J.L., Woschkolup, K., Urrea-Mendoza, E., et al. (2018). Functional Improvements in Parkinson's Disease Following a Randomized Trial of Yoga. *Evid Based Complement Alternat Med* 2018**,** 8516351. doi: 10.1155/2018/8516351.

Vergara-Diaz, G., Osypiuk, K., Hausdorff, J.M., Bonato, P., Gow, B.J., Miranda, J.G., et al. (2018). Tai Chi for Reducing Dual-task Gait Variability, a Potential Mediator of Fall Risk in Parkinson's Disease: A Pilot Randomized Controlled Trial. *Glob Adv Health Med* 7**,** 2164956118775385. doi: 10.1177/2164956118775385.

Volpe, D., Signorini, M., Marchetto, A., Lynch, T., and Morris, M.E. (2013). A comparison of Irish set dancing and exercises for people with Parkinson's disease: a phase II feasibility study. *BMC Geriatr* 13**,** 54. doi: 10.1186/1471-2318-13-54.
